# Flavonoids: Classification, Function, and Molecular Mechanisms Involved in Bone Remodelling

**DOI:** 10.3389/fendo.2021.779638

**Published:** 2021-11-23

**Authors:** Priyanka Ramesh, Rahul Jagadeesan, Saravanan Sekaran, Anuradha Dhanasekaran, Selvaraj Vimalraj

**Affiliations:** ^1^ Centre for Biotechnology, Anna University, Chennai, India; ^2^ Department of Pharmacology, Saveetha Dental College and Hospital, Saveetha Institute of Medical and Technical Sciences (SIMATS), Saveetha University, Chennai, India

**Keywords:** flavonoids, bone, osteoblast, bone remodelling, osteoclast

## Abstract

Flavonoids are polyphenolic compounds spotted in various fruits, vegetables, barks, tea plants, and stems and many more natural commodities. They have a multitude of applications through their anti-inflammatory, anti-oxidative, anti-carcinogenic properties, along with the ability to assist in the stimulation of bone formation. Bone, a rigid connective body tissue made up of cells embedded in a mineralised matrix is maintained by an assemblage of pathways assisting osteoblastogenesis and osteoclastogenesis. These have a significant impact on a plethora of bone diseases. The homeostasis between osteoblast and osteoclast formation decides the integrity and structure of the bone. The flavonoids discussed here are quercetin, kaempferol, icariin, myricetin, naringin, daidzein, luteolin, genistein, hesperidin, apigenin and several other flavonoids. The effects these flavonoids have on the mitogen activated protein kinase (MAPK), nuclear factor kappa β (NF-kβ), Wnt/β-catenin and bone morphogenetic protein 2/SMAD (BMP2/SMAD) signalling pathways, and apoptotic pathways lead to impacts on bone remodelling. In addition, these polyphenols regulate angiogenesis, decrease the levels of inflammatory cytokines and play a crucial role in scavenging reactive oxygen species (ROS). Considering these important effects of flavonoids, they may be regarded as a promising agent in treating bone-related ailments in the future.

## 1 Introduction

Bone is a composite structure that handles a multitude of processes such as preservation of skeletal size, shape integrity, harbouring marrow and controlling mineral homeostasis. Modelling and remodelling form the basis of bone development and maintenance. These are processes that occur throughout the life. The cycle of bone formation and removal is coordinated all over the body but occur at various sites (1). The structure of a bone is the single most complicated organisation handling the calcium phosphorous metabolism in the human system. Numerous cells are involved in this system. Collagen, a triple helix combined with calcium and phosphorous, make up the basic components of the bone, reinforcing the material making up the human skeleton. There are two types of bones – cortical bones which are the solid ones and trabecular bones which have a soft and intricate structure (2). Bones, as we know, are essential for our posture, movement, protection and housing of delicate organs and agility. The structural framework gives us genetic superiority over other species with respect to our ability to perform various tasks like swimming, walking, climbing and many more. *Homo erectus*, as the name suggests, was the first species to ever walk upright on the face of the earth. Since then, humanity has progressed to great lengths of development and evolution. This singularly portrays the importance of skeleton in the supremacy established by human beings. However, the mechanisms of bone formation and modification become very important. Sadly, the truth is that the mechanisms of bone remodelling aren’t clearly laid out yet (3). This gives a great opportunity for researchers to study and understand deeply about the mechanisms in the near future. From time immemorial, study of history has always helped us to correct our mistakes and improve our knowledge of the concerned arena. In this case, several kinds of research on bone diseases in humans and animals have assisted in gaining knowledge on the mechanisms of bone remodelling cycle. The receptor activator of nuclear factor-kB (RANK)/RANK ligand/OPG and canonical Wingless-related integration site (Wnt) signalling are a part of the major signalling pathways. The bone remodelling cycle is regulated by paracrinal secretions such as growth factors, prostaglandins, cytokines and endocrinal secretions such as ergocalciferol, calcitonin, parathormone (PTH), glucocorticoids, thyroxine, estrogen and testosterone (4). Flavonoids, a group of naturally derived compounds with variable phenolic structures, are found in plant foods that are a part of our everyday lives. Flavonoids have many beneficial effects stemming from the significant presence of antioxidant activity, anti-resorptive effects and free radical scavenging capacities (5). They play a prime role in various sectors ranging from nutritional, pharmaceutical to medicinal and cosmetic applications. Research studies have shown that flavonoids assist in lowering the cardiovascular mortality rate and coronary heart disease (6). Flavonoids like Quercetin, Kaempferol, Genistein, Daidzein etc., show healing properties for osteoporosis, a leading cause for joint pain and loss of bone density by regulating osteoblast(OB) and osteoclast (OC) differentiation (7). Soy Isoflavones, in particular, show promising results with antiresorptive activity *via* osteoclast inhibition and promotion of osteoblast differentiation. This is because of their weak binding to the estrogen receptor and a higher affinity towards ERβ when compared to ERα, thus mimicking estrogen. Recent studies also indicate the role of soy isoflavones in activating signalling *via* bone morphogenetic proteins (BMP), thus exhibiting estrogen-independent properties (8). Asian foods have always been rich in flavonoid content, and this might be the probable cause of the increased lifespan of Asian individuals, as they assist in curing many fatal diseases like cancer, cardiovascular diseases and diabetes (8–10). Flavonoids have long been used in Chinese medicine to cure bone fractures, diabetes and many other morbidities (11–14). As many studies highlight, these phytochemicals have a plethora of functions and a huge potential for applications in various fields. Science is yet to divulge into the actualities of molecular mechanisms of flavonoids, and this paper attempts to devise a link between flavonoids and their potential to provide a cure for bone diseases like osteoporosis, inflammation of bone associated with rheumatoid arthritis and periodontal disease, and to give a lucid comprehension of primal flavonoids and the benefits they provide in the systemic metabolism of humans.

## 2 Bone Remodelling

Bone is a complex dynamic structure under continuous remodelling characterised by the resorption of damaged or old bone by the osteoclasts, followed by its’ replacement with the newly formed bone by the osteoblasts. A proper balance between bone resorption and formation is required to maintain a healthy skeleton (15). Bone remodelling tends to become absolutely necessary as it facilitates the primary bone to be replaced by the secondary bone which has higher mechanical strength, removes microfractures and ischemic fractures in bones and at last, assures a correct balance of Ca+/K+ (16). Bone remodelling requires the co-ordinated function of four types of cells namely, bone-lining cells, osteocytes, osteoclasts, and osteoblasts and involves four phases: activation phase, resorption phase, reverse phase and formation phase (17). Osteoclasts are cells sourced from the myeloid, distinctly marked by the presence of multiple nucleus and expression of tartrate-resistant acid phosphatase (TRAP) and the calcitonin receptor (18, 19). The cytokines Colony stimulating factor-1 (CSF-1) and receptor activator of nuclear factor – kappa B(NF-kB) ligand (RANKL) regulate the survival and differentiation of osteoclast precursor cells (1). After differentiation, osteoclasts form an association with the surface of bone through alpha-v beta integrin that transmits signals regulating the organization of the cytoskeleton. The signals thereby activate proto-oncogene tyrosine-protein kinase Src (c-Src), spleen tyrosine kinase (SYK), Guanine nucleotide exchange factor VAV3 Ras homologous GTPases (20). Microscopic trenches are formed on the bone trabeculae surface by secretion of hydrochloric acid and proteases, like cathepsin K (CTSK), into an extracellular lysosomal space to degrade the matrix and mineral parts of the bone (21). Several osteotropic factors such as Interleukin-11 (IL-11), IL-1, PTH and 1,25-(OH)_2_D_3_, indirectly enhance osteoclast formation by stimulation of RANKL on the surface of osteoblasts, followed by RANKL binding RANK on osteoclast precursors. This gives rise to the activation of downstream signalling pathways such as the NF-κB, Ak strain transforming (AKT) pathway, c-Jun N-terminal kinase (JNK) pathway, p38 mitogen activated protein kinase (MAPK), and extracellular signal regulated kinase (ERK) pathway (22–26). The other factors associated with RANK-activated signalling pathways like c-fos, c-src, TNF- Receptor associated Factor 6 (TRAF-6) and Nuclear factor of Activated T-cells (NFATc-1) also play an important role in regulation of osteoclastogenesis (27–29). The formation of osteoclasts and their subsequent activation is limited primarily by various factors, in particular osteoprotegerin (OPG) which plays an inhibitory role by acting as a decoy receptor for RANKL. The homeostasis of RANKL/OPG is a major determinant for the integrity of bone (30).

Neural crest progenitor cells and mesodermal cells give rise to osteoblasts, leading to the differentiation of progenitors into proliferating preosteoblasts, osteoblasts and then into osteocytes. Runt-related transcription factor 2 (RUNX2) is essential for progenitor cell differentiation across the osteoblast lineage (31). During the proliferation of cells, RUNX2 regulates vascular endothelial growth factor (VEGF), osteocalcin (OCN), Receptor activator of nuclear factor kappa-B ligand (RANKL), dentin matrix protein 1 (DMP1) and sclerostin (32). osterix (OSX), insulin-like growth factor (IGF), Bone morphogenetic proteins (BMPs), fibroblast growth factor (FGF), endothelin-1 and PTH regulate differentiation of osteoblasts (33, 34). BMP and PTH are related to activating Wnt signalling pathways (35). The fully differentiated osteoblast is distinguished by coexpression of alkaline phosphatase and type I collagen, both crucial for production of bone matrix and the subsequent mineralization (36). Mature osteoblasts generate mineralization regulators such as osteonectin (ON), OCN, osteopontin (OPN) and RANKL required for osteoclast differentiation. During the end of their lifetime, osteoblasts change into either osteocytes embedded in mineralized matrix or lining cells wrapping the bone surfaces (37). Thus, the homeostasis between bone formation by osteoblasts and bone resorption by osteoclasts, tightly coupled and regulated by various pathways, transcription factors and secreted molecules decide the overall integrity and structure of the bone.

## 3 Bone Diseases

When the cycle of bone remodelling gets disturbed, and the level of osteoclastogenesis exceeds the level of osteoblastogenesis, it weakens the bone resulting in conditions like osteoporosis, periodontitis and rheumatoid arthritis (21).

Osteoporosis is one of the leading causes of bone fractures. There are about nine million fracture incidences worldwide, resulting in a cost of $100 billion. Osteoporotic hip fractures have about 200 million occurrences, and this highlights the great danger that it poses. In first world countries like USA and Europe, even with top-notch medical facilities, 30% of women have osteoporosis, and 40% of post-menopausal women and 30% of men have a high chance of experiencing osteoporotic fracture (38). Sex steroid deficiencies post menopause alter the production of T-cell cytokines which in turn affect the production of RANKL/OPG by the cells of osteoblastic lineage leading to excessive differentiation of osteoclasts and hence excessive resorption. Moreover, pathological conditions involving inflammation increases osteoclastogenesis *via* the production of M-CSF, RANKL, PTHrP, cytokines and prostaglandins (39). An example of this is the overproduction of osteoclasts mediated by IL-6 being a cardinal pathophysiological change in sex-steroid induced osteoporosis (40). Another bone remodelling degenerative disease is periodontitis which involves alveolar bone loss (BL), gingival inflammation, clinical attachment loss (CAL), bleeding, exfoliation of the tooth and periodontal pocketing (41). The disease progression is characterised by excessive production of matrix metalloproteinases (MMPs), leukotrienes, M-CSF, inflammatory cytokines and mediators such as IL-6, IL-1β, TNF-α, prostaglandin E2 (PGE-2) by an over-reactive immune system. The cytokines IL-6 and IL-1β were identified to be the most potent cytokines contributing to bone resorption *via* activation of RANKL, thereby promoting osteoclast activity (42). Rheumatoid arthritis a chronic, systemic, inflammatory autoimmune disorder is characterised by symmetric, erosive synovitis and, in certain cases, extraarticular involvement (43). Bone erosion in RA is typified by the involvement of autoantibodies early in the disease as well as several inflammatory cytokines including TNF-α, IL-6, IL-1β and IL-17 which exert pro-osteoclastogenic effects *via* stimulation of production of RANKL and M-CSF (44–46).

When any disease is subjected to treatment, two parameters have to be primally considered: Selectivity and Therapeutic index (47). In addition to both of these prerequisites, convenience is also important while deciding treatment methods. Convenience refers to the preference of the patients to consume the drugs in particular routes than other routes. Though parenteral routes have many advantages, recipients traditionally prefer the oral route, as it is much less of a discomfort (48). Amongst the prerequisites mentioned above, therapeutic index and convenience are already satisfied as toxicity is almost zero and administration is through oral route. Though the third requirement, selectivity, is not adequate for flavonoids, this can be increased by changing the glucose content associated to give rise to glucoside compounds having higher selectivity, thus making flavonoids better and safe than any other medications present (49).

## 4 Natural Flavonoids

Flavonoids are bioactive compounds belonging to an important class of low molecular weight plant secondary metabolites having a polyphenolic structure. Flavonoids are widely found in fruits, vegetables, herbs, beverages, spices and oils. Hence, they are also known as dietary flavonoids (6, 50). Following terpenoids (30,000) and alkaloids (12,000), the third-largest group of natural products is represented by flavonoids, comprising nearly 10,000 compounds (51). All flavonoids contain 15 carbon atoms in their basic skeleton which are distributed as two six-membered rings and one three-carbon unit linked to them as C6-C3-C6 (51, 52). The 3-carbon unit bridging the phenyl groups usually cyclizes with oxygen to form a third ring. This core structure is called 2-phenylbenzopyranone (53). Flavonoids are most often associated with sugar in the conjugated form to be O-glycosides or C-glycosides. They can also exist as aglycones (54). The glycosides are normally attached to position 3 or 7, with the most common carbohydrates occupying those positions being D-glucose, L-rhamnose, gluco-rhamnose, galactose or arabinose (52). The other factors pertaining to the varied chemical nature of the flavonoids include patterns of hydroxylation, conjugation between aromatic rings, methoxy groups, and other substituents such as sulphates and prenyl groups (51, 55). Flavonoids have been known to exhibit a broad spectrum of pharmacological and biochemical reactions associated with health promoting effects. Examples of such therapeutic properties are anti-inflammatory, hepatoprotective, anti-mutagenic, anti-oxidative, anti-neoplastic, anti-viral, anti-microbial, anti-helminthic, anti-allergic, anti-hormonal, anti-thrombotic, differentiation and apoptotic effects (6, 7, 56). Numerous *in-vitro* studies have shown flavonoids capacity in modulating the key cellular enzymes. Modulation of these enzymes, in turn, affect the important cellular pathways which regulate cell division and proliferation, inflammatory and immune responses, detoxification and platelet aggregation (57). Flavonoids act as potential metal-chelators and free radical scavengers. They neutralise free radicals by donating electrons from their conjugated double bonds and groups *via* resonance, thus acting as natural anti-oxidants (51, 56). Recent studies have discovered the connection between flavonoids and the regulation of bone metabolism. This property is being studied, to use flavonoids as a possible therapy in the future, for the treatment of osteoporosis (7).

## 5 Classification of Flavonoids

Flavonoids can be broadly categorised into three groups: the bioflavonoids, the iso-flavonoids (phytoestrogens) and the neo-flavonoids (white flavonoids) (50). The variations in the different classes and subclasses of flavonoids are attributed to factors such as the degree of unsaturation, the carbon of the C ring to which the B ring is attached, degree of hydroxylation, degree of oxidation, glycosylation pattern and other substitutions (51).

### 5.1 Iso-Flavonoids

In iso-flavonoids the B ring is attached to position 3 on the C-ring (6). Iso-flavonoids structurally resemble 17-beta estradiol and bind to oestrogen receptors. Hence, they are also known as phyto-oestrogens. Depending on the endocrine estrogenic levels, they can act as either agonists or antagonists (8, 58). Iso-flavonoids possess tremendous potential to fight various diseases including amelioration of osteoporosis and cardiovascular disease, prevention and treatment of hormone-related cancer, treatment of menopause symptoms and other age related diseases (59). The major sources of isoflavones are the leguminous plants belonging to the family Fabaceae/Leguminosae. Other sources include red clover, red wine, germs of alfalfa and linseed, with red clover containing the highest amount of phyto-estrogens (58, 60). Some examples of isoflavones are Genistein, daidzein, glycitein, biochanin A and formononetin (60).

### 5.2 Neo-Flavonoids

Flavonoids in which the B-ring is attached to position 4 of the C-ring are known as neo-flavonoids (NFs). The first neoflavone to be isolated was calophyllolide from Calophyllum inophyllum seeds (6). NFs have been categorised into two broad groups namely, the 4-phenylcoumarins (dalbergin group) and the diphenyl allyl compounds (latifolin group). They are distributed in a wide range of plants belonging to families Fabaceae, Clusicaea, Leguminosae, Rubiaceae, Passifloraceae, Thelypteridaceae and Polypodiaceae. The most abundantly found neo-flavone is Dalbergin isolated from various plants of the genus Dalbergia. NFs exhibit several therapeutic properties which include anti-allergic, anti-inflammatory, anti-osteoporotic, antimicrobial and anti-oxidant (61).

### 5.3 Bio-Flavonoids

Those class of flavonoids in which the B-ring is attached to position 2 of the c-ring are called as bio-flavonoids. They can be further subdivided into different subclasses depending on the structural features of the C-ring. These subclasses are flavones, flavonols, flavonones, flavan-3-ols/catechins, anthocyanidins and chalcones (6). Flavonols are the most common and largely occurring flavonoids in the plant kingdom. Examples of major dietetic flavonoids are quercetin, kaempferol, fisetin, isorhamnetin and myricetin, with quercetin being one of the most abundant flavonoids of the human diet (62). Flavones are majorly found in foods such as celery, lettuce and capsicum peppers (50). The main flavones of the human diet include apigenin and luteolin (62). Catechins, otherwise known as flavan-3-ols possess a hydroxyl group in C3 of C-ring (a dihydro-pyran heterocycle). Catechins, gallocatechin, epigallocatechin, epicatechin and gallate are a few compounds that fall under this category. A variety of fruits, vegetables and plant-based beverages contain abundant concentrations of catechin. Green tea is the main dietary source (63). Flavanones have a basic skeleton of 2-phenylbenzopiran-4-one. They play a vital role in regulating the metabolic pathways of other flavonoids (64). They are found in almost all citrus fruits and are responsible for their bitter taste. Hesperitin, naringin and eriodictyol are a few examples of this subclass (6). Anthocyanidins are another subclass of bio-flavonoids that are water-soluble and are found in the leaves, stems, roots, flowers and fruits of all higher plants. They are responsible for the red, purple and blue colour of certain fruits, which vary depending on the pH. Cyanidin, peonidin, pelargonidin, delphinidin, petunidin and malvidin are the most prevalent compounds (53). The last subclass, chalcones are open-chain flavonoids. They consist of two aromatic rings A and B joined by a 3-carbon a,b-unsaturated carbonyl group. Leguminosae, Asteraceae and Moraceae are the three families that contain the largest number of natural chalcones. Examples of chalcones include naringenin chalcone, isoliquiritigenin, phloretin, licodione, echinatin etc (65).

#### 5.3.1 Quercetin

**Figure 1:**
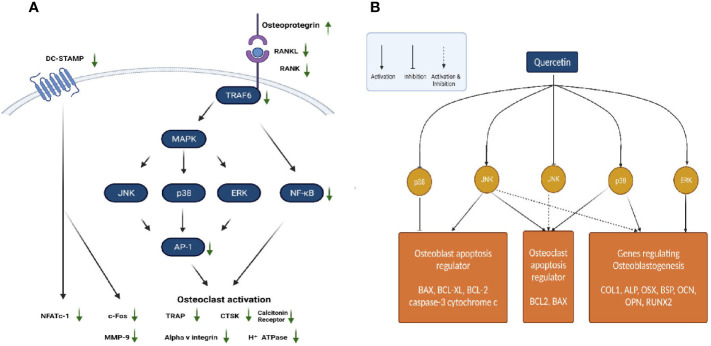
**(A)** Regulation of RANK/RANKL/OPG system by quercetin. **(B)** Actions of quercetin on JNK, ERK and p38 MAPK pathways.

Quercetin is one of the most important and widely studied dietary bioflavonoids. It is ubiquitously found in fruits and vegetables (66). For several years in China, Quercetin and its derivatives have been used in the treatment of osteoporosis because of their natural anti-oxidant property (67). Quercetin regulates various pathways involved in maintaining bone homeostasis such as the RANK/RANKL/OPG System, MAPK signalling, apoptotic pathway, canonical Wnt/β Catenin signalling, BMP and transforming growth factor (TGF-β) signalling (**Figures 1A, B**). Further quercetin exhibits anti-oxidative, anti-inflammatory and angiogenic properties through which it maintains a balance between osteoblastogenesis and osteoclastogenesis (66). Prouillet et al., showed that, in MG-63 human osteoblasts, quercetin had a stimulatory effect on alkaline phosphatase (ALP) activity in the range of 1-50 mM without any significant cytotoxic effects. Quercetin-induced ALP activation requires the ERK pathway and rapidly stimulates it, because inhibition of this pathway by the MEK inhibitor PD 98059 reduced the enhancing actions of quercetin. Moreover, the direct role of ER involved in the effect of quercetin was shown by the fact that ER antagonist ICI 182780 prevented quercetin-induced increase in ALP activity (68). While the previous study showed involvement of ER, another study on the effects of quercetin pretreatment on osteogenic differentiation and proliferation of Human Adipose Tissue Derived Stromal Cells (hADSC) indicated an ER-independent mechanism (69, 70). In mouse monocyte/macrophage cell line RAW264.7, quercetin and quercetin-3-O-glucoside (Q3G) were found to decrease the number of RANK-L-induced TRAP positive multi-nucleated osteoclast cells significantly in dose dependent manner. Treatment with quercetin suppressed the expression of osteoclast related genes such as the calcitonin receptor (CTR), CTSK, MMP-9 and NFATc1. NFATc1 is a master regulatory transcription factor of osteoclast differentiation regulated by RANK-L *via* activator protein-1 (AP-1) and NF-kB (71, 72). Actin-ring formation, which is important for bone resorption in osteoclast-like mononucleated cells (OCLs) was disrupted by quercetin. This suggests a possible role of quercetin in regulating the signal transducing molecules involved in actin-ring formation: p60 c-src tyrosine kinase, phosphoinositide-3-kinase (PI3K), GTP-binding proteins (GTP-BP) and protein kinase A (PKA) (73). IL-17 is an osteoclastogenic inflammatory cytokine promoting the production of other destructive cytokines such as the macrophage migration inhibitory factor (MIF), tumour necrosis factor-alpha (TNF-α) and RANK-L which in turn increase reactive oxygen species (ROS) and osteoclastic differentiation in rheumatoid arthritis (RA). IL-17-stimulated RA-fibroblasts-like synoviocytes (RA-FLS), when treated with quercetin decreased the production of RANK-L, TNF-α, IL-6 and IL-8. Quercetin decreased the IL-17-induced phosphorylation of mammalian target of rapamycin (mTOR), ERK and NF-kB in RA-FLS, whereas it increased the IL-17-induced phosphorylation of AMP-activated protein kinase (AMPK). Since AMPK is known to counteract and inhibit mTOR signalling, the effect of quercetin on AMPK activation suppresses mTOR and induces apoptosis in osteoclasts (74). MC3T3-E1 cells, treated with Lipopolysaccharide (LPS), a pro-inflammatory glycolipid suppressed the m-RNA and protein expression levels of ALP, RUNX2, OSX and OCN, thus inducing apoptosis and inhibiting the differentiation of osteoblasts *via* the JNK pathway. Quercetin reversed this condition by increasing the phosphorylation of ERK-1/2, which inhibited the induction of apoptosis by p38 MAPK and JNK. Further, quercetin upregulated the expression of anti-apoptotic proteins B-cell lymphoma-2 (BCL-2) and BCL-XL, while it downregulated the apoptotic proteins caspase-3, BCL-2 associated X apoptosis regulator (BAX) and cytochrome c (75). In osteoblasts isolated from foetal rat calvaria quercetin aglycone upregulated the m-RNA and protein levels of three anti-oxidant genes heme oxygenase-1 (HO-1), γ-glutamate cysteine ligase catalytic subunit (GCLC) and catalase. However, it did not upregulate Nuclear factor erythroid 2–related factor 2 (Nrf-2), the transcription factor of these three genes. Quercetin also downregulated the phosphorylated levels of ERK1/2 and NF-kB, which suggests an anti-inflammatory response associated with the activation of anti-oxidant genes (76). This is in contrast to the studies on MC3T3-L1 osteoblasts and MG-63 osteosarcoma cells (68, 77). Further studies are required to confirm the exact role of ERK1/2, NF-kB p65 and Nrf-2 in mediating the anti-oxidative responses (76). Zhou et al., investigated the effect of quercetin on angiogenesis and found that it increased the expression of angiogenic factors VEGF, angiopoietin 1 (ANG-1), basic fibroblast growth factor (bFGF) and TGF-b, ultimately leading to bone regeneration (66). Another pathway regulating bone homeostasis is the Wnt/b-catenin pathway. Pre-treatment of MC3T3-E1 cells with quercetin increased the protein levels of Wnt3 and β-catenin, which is responsible for osteoblast differentiation (75). One of the mechanisms by which TNF-α suppresses osteoblastogenesis is by inhibiting the activation of SMAD signal transduction by TGF-β and BMP-2. The effect of quercetin in this case, only added to the inhibitory effect of TNF-α, rather than suppressing it. Thus, the overall effect of quercetin on bone formation involves complex competing pathways which may depend on the dose and the concentrations of cytokines and growth factors prevailing in the micro-environment (72).

#### 5.3.2 Kaempferol

**Figure 2:**
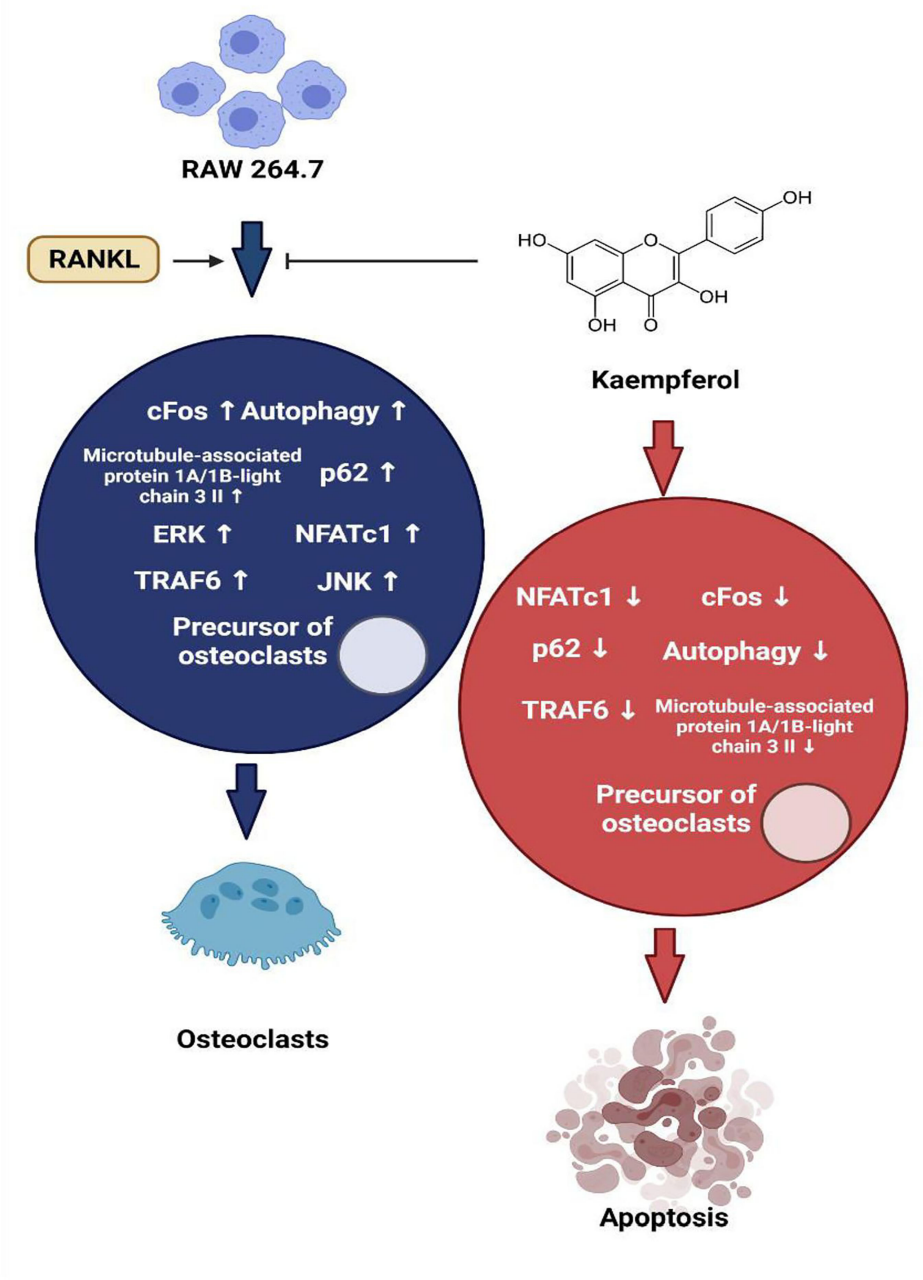
Regulation of autophagy and apoptosis of osteoclasts by kaempferol *via* degradation of p62/SQSTM1.

Kaempferol and its derivatives are natural bioflavonoids enriched in fruits and vegetables and are used as nutraceuticals. Kaempferol possesses various medicinal properties some of which are directly associated with bone-sparing effects (78). Both adipocytes and osteoblasts are differentiated from multipotential mesenchymal stem cells in bone marrow. During the process of ageing, there is a reciprocal increase in adipogenesis and decrease in osteogenesis in the bone marrow, which has to be inhibited and reversed to treat bone diseases such as osteoporosis. The *in vitro* studies of Ritu et al., showed that kaempferol inhibited the differentiation of bone marrow mesenchymal stem cells(BMSCs) to adipocytes, whereas it stimulated increased osteoblast differentiation (79). This is supported by the fact that kaempferol downregulated the LPS-induced expression of lipid-anabolism genes (sterol regulatory element binding protein-1c [SREBP-1c], fatty acid synthase [FAS] and peroxisome proliferated activated receptor-gamma [PPAR-g]) in BMSCs (**Figure 2**). On the contrary, it promoted the expression of genes involved in lipid catabolism (carnityl palmitoyl transferase [CPT-1], PPAR-α and acetyl CoA carboxylase [ACC]), thus preventing adipogenesis (80). Kaempferol treatment of BMSCs increased the expression of important downstream regulatory proteins in the mTOR pathway, which suggests its involvement in the differentiation of osteoblasts. The role of mTOR in osteogenesis was validated by Zhao et al., where treatment BMSCs with a specific inhibitor of mTOR called rapa, resulted in decreased levels of osteogenic activity. However, several other studies exhibit controversies over the role of mTOR in bone formation (81). In mouse calvarial osteoblast cell line MC3T3, kaempferol inhibited the TNF a-induced signalling in osteoblasts and thereby reduced the secretion of osteoclastogenic cytokines interleukin-6 (IL-6) and monocyte chemoattractant protein-1 (MCP-1). It also blocked the TNFa-induced nuclear translocation of NF-kB, a transcriptional regulator of MCP-1. Further, kaempferol antagonised the RANKL induced differentiation of RAW264.7 cells to osteoclasts by inhibiting c-Fos expression, an immediate early oncogene, which is indispensable for osteoclastogenesis (82). In LPS treated BMSCs, kaempferol reversed the downregulation of expression of chondrogenic markers SRY-Box Transcription Factor 9 (SOX-9), COL-2 and Aggrecan and strongly elevated their levels. Besides, kaempferol caused a significant decrease in the levels of matrix metallo-proteinase-3 (MMP-3), MMP-13, ADAM metallopeptidase with thrombospondin Type 1 Motif-4 (ADAMTS-4), ADAMTS-5. The inflation of pro-inflammatory cytokines IL-6, IL-1β, inducible nitric oxide synthase (iNOS) and TNF-α induced by lipopolysaccharide (LPS) was reduced by kaempferol, while it increased the level of anti-inflammatory cytokine IL-10 (80, 83). The LPS-induced activation of NF-kB was also inhibited by kaempferol, as was shown by the reduced nuclear staining of p-65 (80). Treatment with kaempferol of ATDC5 cells, led to a marked increase in the mRNA levels of genes encoding COL-2 and COL-10, which are markers of fully differentiated chondrocytes. Also, kaempferol induced the activation of ERK and p38 MAP kinase pathway. Further, it promoted the expression of BMP-2 and BMP-4, thereby suggesting that stimulation of chondrogenesis occurs *via* BMP-2 signalling pathway in ATDC-5 cells (84). Treatment with 8-prenyl kaempferol, a prenyl flavonoid on MC3T3-E1 cell line, regulated osteoblast differentiation *via* BMP-2 signalling pathway, which subsequently triggered SMAD1/5/8. This led to the activation of the transcription factor RUNX2 which promoted bone mineralization by regulating the expression of COL-1, OPN and ON (85). Kaempferol induced luciferase activity in rat primary osteoblasts transfected with pERE-Luc and also triggered phosphorylation of ER-a, which suggested that kaempferol acts *via* ER activation. This was confirmed when pre-treatment with ICI 182,780 completely blocked the kaempferol-induced pERE-Luc activity. Additionally, kaempferol upregulated ALP activities and the transcription of several bone differentiation marker genes such as the COL1A1, ON, OCN, RUNX2 and OSX (86). A study by prouillet et al., on MG-63 human osteoblastic cell strain demonstrated that kaempferol induced increase in ALP activation involves the ERK pathway. This was shown by incubating the cells with PD 98059, an inhibitor of ERK pathway, which reduced the stimulatory effects of kaempferol on ALP. ICI 182780, a pure anti-estrogen, inhibited ERK activation and reduced the levels of ALP in kaempferol treated cells which shows that kaempferol activates ERK pathway *via* the ERs (69, 85). MAP kinase activation *via* a non-genomic action of ER can lead to downstream modulation of the transcription factor AP-1 which has been predicted to have a binding site on the promoter of the ALP gene. This transcription factor can act as a possible link between rapid ERK activation and increased ALP activity (69). Pretreatment with kaempferol of MC3T3-E1 cells exhibited a marked reduction in antimitochondrial antibody (AMA) induced-cell damage by preventing mitochondrial membrane potential dissipation, complex IV inactivation, [Ca^2+^]_i_ elevation, and ROS production. Kaempferol induced the activation of AKT, PI3K and cAMP response binding element protein (CREB) inhibited by AMA, which are known to be involved in osteoblast-like cell proliferation and differentiation (87). RANK-L induced differentiation of RAW 264.7 cells to osteoclasts was shown to be inhibited by kaempferol by suppressing the expression of osteoclastogenic factors TRAF6, NFAT-c1, and c-Fos. Osteoclastogenesis was also suppressed by inhibiting autophagy related factors beclin-1 and sequestosome 1 (p62/SQSTM1) (83, 88). Furthermore, in dexamethasone-induced rat calvarial osteoblasts, kaempferol decreased osteoblast apoptosis by inducing expression of the anti-apoptotic gene BCL-2 and suppressing BAX, a pro-apoptotic gene (78).

#### 5.3.3 Icarin

**Figure 3:**
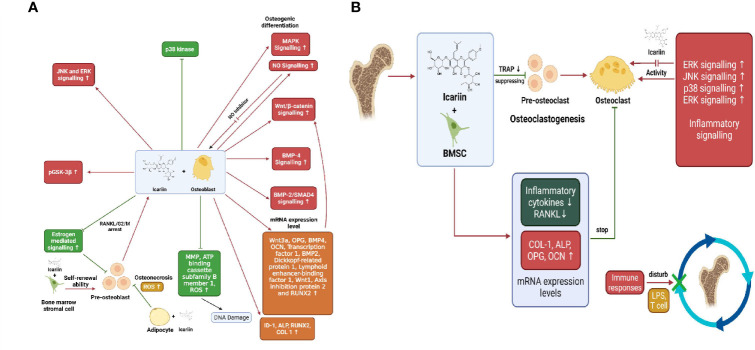
**(A)** Osteogenic effects exerted by icariin through promotion of osteoblastogenesis and inhibition of adipocyte differentiation from pre-osteoblasts. **(B)** Inhibition of bone resorption by icariin *via* inhibition of osteoclast-related genes and pathways.

Icariin is the main active prenylated flavonol glycoside isolated from the herb Epimedium pubescens. It has been widely used for several centuries in Chinese herbal medicine and is known to possess “bone strengthening” properties (89, 90). Naturally isolated icariin is becoming an interesting alternative in the prevention and treatment of bone diseases (90). It has been known to enhance osteoblastic differentiation and proliferation, inhibit bone resorption, and induce apoptosis of osteoclasts (89). Pre-osteoblastic MC3T3-E1 cells treated with icariin upregulated the levels of osteogenic markers RUNX2, OCN, BSP and ALP in a dose dependant manner. Besides, Icariin was effective in upregulating RUNX2, BSP and OCN levels in mouse primary osteoblasts as well. mRNA expression levels of inhibitor of DNA binding-I (Id-1), a transcriptional target of BMP/SMAD signalling was increased in MC3T3-E1 cells when treated with icariin, whereas expression of RUNX2 m-RNA was upregulated in both MC3T3- E1 cells and POBs. This suggests the involvement of BMPs and RUNX2 signalling in osteogenesis induced by icariin (91, 92). In adult female osteoblast-like cells, icariin caused a significant increase in ALP activity and nitric oxide (NO) levels followed by increased proliferation and mineralisation of osteoblasts (**Figure 3A**). NO is known to exhibit inhibitory effects on bone resorption by suppressing osteoclasts activity and precursor recruitment connected to iNOS activity. Moreover, icariin treatment increased BMP-2/SMAD protein expression as well. Both NO and BMP-2/SMAD activate the transcription of RUNX2 gene, thereby regulating bone homeostasis. Icariin also attenuated caspase-3 activity in the osteoblast-like cells on the 28^th^ day of treatment with icariin, thereby exhibiting its anti-apoptosis effect (93). Sheng et al. demonstrated that treatment with icariin upregulated OCN synthesis, ALP activity, calcium deposition and collagen synthesis in BMSCs, thus promoting osteogenic differentiation. Additionally, icariin increased the expression levels of marker genes and proteins namely RUNX2, OSX, BMP-2 and IGF in osteogenic cultures. Ming et al. and Huang et al. found that icariin inhibited osteoclastogenesis induced by RANKL and M-CSF in mouse bone marrow culture and inhibited bone resorption by stimulating apoptosis of mature osteoclasts (89). In the study of Wu et al., on BMSCs, it was found that ERK, p38 and JNK signalling pathways were all phosphorylated indicating their participation in osteoblast proliferation, differentiation and mineralisation. Blocking these three pathways significantly inhibited ALP activity and expression of COL1, OPN and OCN. Besides, icariin treatment has also been reported to instigate osteogenic differentiation of BMSCs through the activation of PI3K–AKT–eNOS–NO–sGC–cGMP–PKG signalling pathway (94). Icariin caused significant inhibition of NF-kB activation in RANKL-induced RAW264.7 cells by degradation of nuclear factor of kappa light polypeptide gene enhancer in B-cells inhibitor, alpha (IkB-a) (**Figure 3B**). RANKL-induced expression of downstream regulatory factors c-Fos and NFATc1 were decreased after treatment with icariin, which in turn reduced the levels of target osteoclastogenic proteins such as CTSK and TRAP (95). Treatment with icariin on bone mesenchymal stem cells upregulated the expression of osteogenic genes RUNX2, ALP, and COL1 and decreased the expression levels of adipogenic genes —PPARγ, fatty acid binding protein-4 (Fabp4), and adipsin, thus inhibiting the differentiation of BMSCs into adipocytes. Icariin promoted the phosphorylation of Glycogen synthase kinase-3β (GSK-3b) and elevated the levels of active b-catenin in the nucleus of BMSCs. Inhibition of the Wnt signalling pathway brought down the phosphorylation of GSK-3b, caused degradation of b-catenin and upregulated the expression of adipogenic genes, thus confirming the intervention of the Wnt pathway in the differentiation of BMMSCs (92, 96). Recently it was found that cyclin D1, a mitogenic signal sensor that pushed cells from G0 phase into the proliferative cycle, was significantly increased in icariin treated BMSCs (92). Iron overload and accumulation in post-menopausal women and elderly men has been found to be linked to bone metabolism abnormalities like osteopenia, osteomalacia and osteoporosis. Treatment with icariin reversed the iron-overload-induced elevation of ROS and mitochondrial dysfunction caused by the collapse of mitochondrial membrane potential. Thus, icariin attenuated the increase in osteoclasts differentiation and promoted osteoblasts proliferation and differentiation in iron-overloaded MC3T3-E1 osteoblasts (97). Icariin inhibited hypoxia induced apoptosis in neonatal rat calvarial osteoblasts. It reduced the expression levels of caspase-3 and upregulated the mRNA expression levels of BCL-2, thereby inhibiting apoptosis. Also, icariin supplementation diminished the intracellular malondialdehyde (MDA) levels and ROS production, while increasing the activity of SOD, anti-oxidant enzyme to ameliorate the hypoxia induced stress (98). In the LPS-induced osteoclastogenesis model, icariin treatment reduced the LPS-induced activities of osteoclast differentiation marker protein TRAP and regulator of bone resorption- acid phosphatase (ACP). Moreover, icariin suppressed the LPS-induced RANKL expression, whereas it elevated the LPS-inhibited expression of OPG, an osteogenic marker. In addition, icariin could inhibit the synthesis of osteoclastogenic pro-inflammatory cytokines such as IL-6 and TNF-a. Alongside this, icariin reduced prosteoglandin-E2 (PGE-2) production by obstructing synthesis of cyclo-oxygenase -2 (COX-2), therefore inhibiting bone resorption (99). Considering the limitations of animal models in determining the therapeutic efficacy and pharmacological properties of icariin and derivatives, further verification using mammalian models, primates and human clinical trials is required (90).

#### 5.3.4 Myricetin

**Figure 4:**
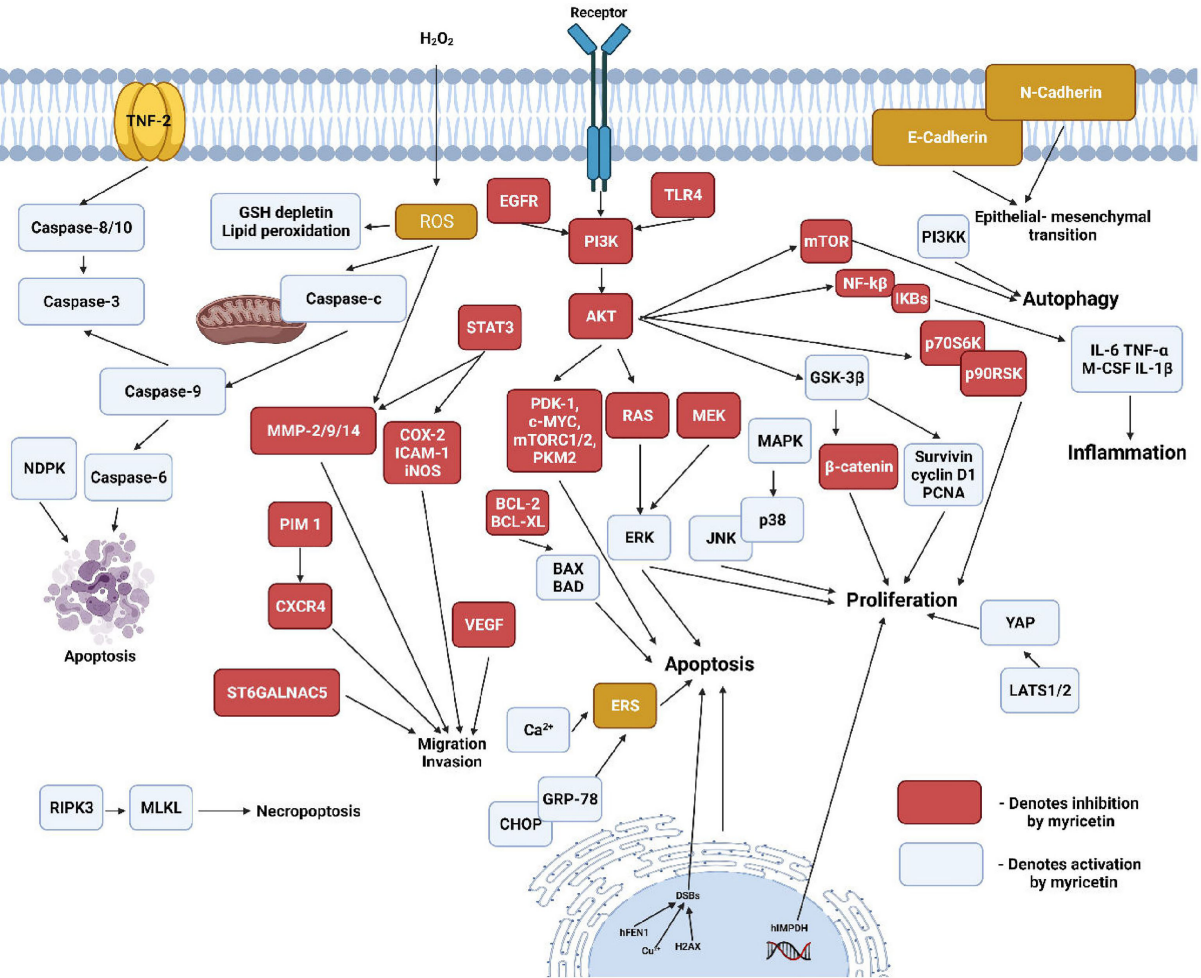
Regulation of cellular pathways by myricetin. (Nucleoside diphosphate kinase-NDPK, Receptor-interacting serine/threonine-protein kinase 3-RIPK3, Mixed lineage kinase domain-like-MLKL, C-X-C chemokine receptor type 4- CXCR4, ST6 N-Acetylgalactosaminide Alpha-2,6-Sialyltransferase 5- ST6GALNAC5, Phosphoinositide-dependent kinase 1-PDK 1, Pyruvate kinase M2-PMK2, Toll-like receptor 4- TLR4, CCAAT/enhancer binding protein homologous protein-CHOP, Glucose-regulated protein 78-GRP-78, Endoplasmic reticulum stress-ERS, Proliferating cell nuclear antigen-PCNA, Yes-associated protein-YAP-1, Large tumour suppressor kinase-1/2- LATS1/2, p90 ribosomal S6 kinase-p90RSK, Ribosomal protein S6 kinase beta-1-p70S6K).

Myricetin belongs to a subclass of bio-flavonoids called flavonols. It is majorly found in berries, fruits, vegetables, medicinal herbs and tea plants (100, 101). Myricetin is known to possess antioxidant, anti-inflammatory, antimicrobial, anti-viral, antioxidative, anti-tumorigenic and antiallergic properties. Recent studies also provide evidence for myricetin exhibiting osteo-protective properties and inhibiting osteo-clastogenesis (102, 103). Huang et al., demonstrated that myricetin treatment suppressed RANKL-induced differentiation of mouse macrophage RAW264.7 cells into characteristic TRAP-positive multinucleated osteoclast-like cells (OCL). The hypothesis that impairment of osteoclast differentiation would also result in the inhibition of osteoclast bone resorption was confirmed by bone resorption assay, which showed complete bone resorption activity at myricetin concentrations ≥50 mm (100). In the Titanium particle-induced mouse calvarial osteolysis model, myricetin disrupted the RANKL-induced F-actin ring formation, a characteristic feature of mature osteoclasts responsible for bone resorption. It also decreased the RANKL-induced expression of osteoclastogenic markers TRAP, CTR, CTSK, V-ATPase-d2, c-Fos, and NFATc1 (**Figure 4**). Further, Myricetin inhibited the production of pro-inflammatory cytokines TNF-a and IL-1b, thereby suppressing the NF-kB pathway and MAPK pathways (p38, JNK1/2, and ERK1/2) responsible for osteoclast formation and bone resorption (104). Ying et al., showed that treatment with myricetin elevated the serum OCN and ALP levels in rats with streptozotocin-induced diabetic osteoporosis. The levels of serum anti-oxidants SOD and catalase were also increased in response to addition of myricetin (105). In human chondrocytes, myricetin reduced the levels of IL-1b stimulated inflammatory mediators and cytokines such as PEG-2, COX-2, iNOS, IL-6 and TNF-a as well as the elevated levels of MMPs, thereby inhibiting extracellular matrix (ECM) degradation and promoting generation of COL-2. Regulation of these mediators was associated with the repression of NF-kB pathway by the activation of Nrf2/HO-1 with a possible mediation of the PI3K/AKT pathway (106). Pre-treatment of human gingival fibroblasts with myricetin suppressed the LPS-induced expression of MMP-1, MMP-2 and MMP-8. RANKL-stimulated RAW264.7 cells when pre-treated with myricetin, exhibited reduced phosphorylation of p38 and ERK pathways, inhibited phosphorylation of c-Src and impeded the degradation of IkB-a. Moreover myricetin showed inhibitory effects on the m-RNA expression of osteoclastogenic markers such as TRAP, c-FOS, CTSK and NFATc-1 (107). Myricetin exhibits protective effects against 2-deoxy-D-ribose induced oxidative damage in MC3T3-E1 cells by decreasing the levels of protein carbonyl, advanced oxidation protein products, and MDA. Besides it elevated the levels of ALP activity, collagen content, calcium deposition, OCN and OPG in the presence of 2-deoxy-D-ribose (108). In human bone marrow stromal cells (hBMSCs), myricetin upregulated the levels of m-RNA expressions of osteogenic markers OCN, COL-1, ALP and RUNX2. Apart from that, myricetin triggered the Wnt/b-catenin pathway and upregulated the expression of several downstream genes such as T-cell factor-1(TCF-1) and lymphoid enhancer factor-1 (LEF-1) (109). Hsu et al., showed that treatment with myricetin on the conditionally immortalized human fetal osteoblastic cell line (hFOB) and the human osteosarcoma cell line MG-63, caused a significant upregulation of BMP-2, which in turn increased the phosphorylated levels of SMAD 1/5/8 and p38, one of the MAPK pathways (110). The effects of myricetin on Dexamethasone(DEX) treated MC3T3 cells revealed that, it ameliorated the DEX-induced inhibition of bone formation markers namely RUNX2, BSP, OPN, OCN, COL1A1 and ALP. Besides myricetin promoted matrix mineralisation *via* the ERK signalling pathway and downregulated TRAP activity and C-terminal telopeptide of type I collagen (CTx) in DEX treated cells (111). Pre-treatment with myricetin on MG-63 cells, reduced the synergistic effect of IL-1b and TNF-a on anti-Fos immunoglobulin-M (IgM) mediated apoptosis of osteoblasts, thereby attenuating the activation of apoptotic proteins caspase-8 and caspase-3, and upregulating the levels of the anti-apoptotic protein FLICE inhibitory protein (FLIP) (112). Overall, myricetin has proven to exhibit osteogenic properties and further studies are required to use it as a therapeutic agent against bone diseases.

#### 5.3.5 Naringin

**Figure 5:**
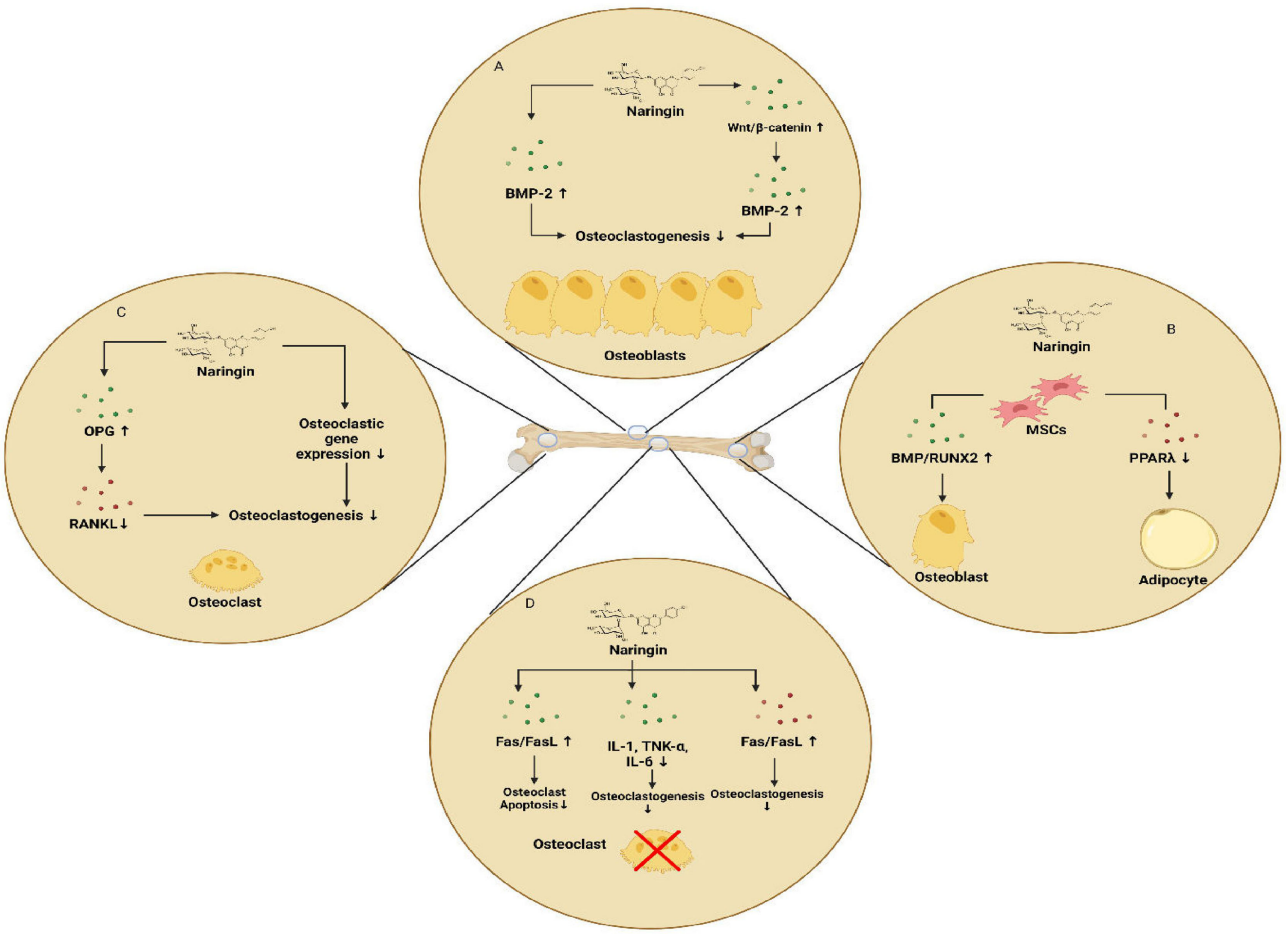
Effects of naringin on bone **(A)** naringin-induced upregulation of osteoblastogenesis *via* regulation of BMP-2 and Wnt/β-catenin pathways **(B)** upregulation of osteoblastogenesis and downregulation of adipogenesis **(C)** inhibition of osteoclastogenesis and osteolysis by naringin mediated by the inhibition of RANK/RANKL interaction **(D)** inhibition of bone resorption by inducing apoptosis of osteoclasts and reducing inflammatory cytokines that induce osteoclast formation.

Naringin, a polymethoxylated flavonoid glycoside, is an active ingredient of citrus fruits and Chinese herbal medicine. It possesses several pharmacological effects, including bone-protective properties. Zhu et al. demonstrated that naringin exhibits anti-osteoporosis property in a fashion similar to estrogen, by binding to the estrogen receptors. This might replace estrogen-replacement therapy in clinical use (113, 114). In the study of Li et al., naringin promoted the osteogenic proliferation and differentiation of BMSCs and also exhibited a 5-7 day delay between the start of naringin treatment and the burst of ALP expression. This suggested a delayed differentiation pattern of the BMSCs in response to naringin treatment (114). In human amniotic fluid stem cells (hAFSCs), naringin was shown to upregulate ALP activity and calcium deposition in a dose dependent manner. Naringin significantly promoted the expression of osteogenic marker genes including ALP, OPN and COL-1 as well as the osteoclastogenesis-inhibition marker gene OPG, thus enhancing the osteogenic differentiation of hAFSCs (**Figure 5**). This differentiation was shown to be regulated *via* the BMP and Wnt/b-catenin pathways involving BMP-4, RUNX2, b-catenin and cyclin D1 (115). Further, naringin induces the apoptosis of osteoclasts *via* inhibition of activation of the death receptor pathway (Fas, TNF) or mitochondrial apoptosis pathway. In the study conducted by Li et al., it was confirmed that naringin could downregulate the mRNA expression levels of the pro-apoptotic marker gene BCL-2 and downregulate the expression levels of the anti-apoptotic marker gene BAX (116). Treatment of RAW627.4 cells with naringin abrogated RANKL induced formation of TRAP positive osteoclast cells. Additionally, naringin attenuated the gene expression levels of osteogenic markers such as CTSK, CTR and TRAP as well as osteoclastogenic fusion genes including dendritic cell-specific transmembrane protein (DC-STAMP), and V-ATPase d2 (d2). Further, naringin suppresses the RANKL induced activation of NF-kB *via* inhibition of degradation of IkB and suppresses the activation of ERK pathway as well (117). Recent studies have shown naringin being an HMG-CoA reductase inhibitor, might possibly promote BMP-2 expression and induce bone formation, suggesting the possible involvement of mevalonate pathway. In co-cultures of osteoblasts and bone marrow cells, naringin suppressed the IL-1 induced osteoclastogenesis (118). Naringin may also possess the ability to downregulate the expression of PPARγ in BMSCs, thus reducing adipogenesis and promoting bone formation. In addition, naringin inhibited the mRNA expression of osteoclastogenic markers including RANK, TRAP, MMP-9 and NFATc1, whereas it upregulated c-Fos expression in RAW627.4 cells (119). Li et al., showed that increased levels of SOD, catalase and MDA in dexamethasone (DEX)-treated-inflammatory bowel disease (IBD) rats were significantly reduced by the intervention of naringin (120). The experiments of Wu et al., revealed that naringin induced osteoblast proliferation, differentiation and maturation in cultured osteoblasts. Besides, in MC3T3-E1 osteoblastic cells, the stimulatory effects of naringin on the expression of BMP-2 was found to involve the activations of PI3K, AKT, c-Fos/c-Jun and AP-1 pathways. Furthermore, it was found that the osteo-protective effects of naringin on UMR-106 cells were attributed to its positive effect on the Wnt/b-catenin pathway *via* AMPK and AKT signalling (121, 122). Kanno et al., demonstrated that naringin inhibited the LPS-induced production of NO and the expression of inflammatory gene products such as TNF-a, IL-6, iNOS, COX-2 and the transcriptional activity of NF-kB. Suppression of these pro-inflammatory cytokines which are the positive regulators of osteoclastogenesis *via* the inhibition of NF-kB might result in the inhibition of osteoclastogenesis and bone resorption (123). Further, naringin promoted angiogenesis and neovascularization during fracture callus formation in murine osteoporotic models, likely by regulating the expression of VEGF in osteocytes (119). Naringin’s diverse effects on bone indicate its potential in the treatment and prevention of many common orthopaedic conditions. Naringin strongly reduces osteoclastogenesis, inflammation, and adipogenesis and promotes osteoblastic differentiation from progenitor cells for the maintenance and preservation of both cartilage and bone. However additional research is required to assess the ways in which the pharmacokinetic properties of naringin can be improved, in order to optimize its therapeutic effects.

#### 5.3.6 Daidzein

Daidzein is a phytoestrogen belonging to the iso-flavonoid group and abundantly found in soy products. Considering the fact that daidzein can bind to estrogen receptors a and b and have estrogenic effects, they can be used as an alternative to estrogen replacement therapy (124). Osteoblast cell cultures treated with exhibited enhanced osteoblast viability and induced their differentiation from osteoprogenitors to terminally differentiated osteoblasts. Moreover, daidzein increased the ALP activity, OCN synthesis and the mRNA expression levels of BMP-2 in primary osteoblast cell cultures (125). Exposure of porcine osteoblasts to daidzein increased the nuclear levels of the osteogenic transcription factor RUNX2 that was blocked by ICI 182,780. Daidzein also caused a heightened secretion of OPG in the medium of porcine control OB, while it decreased the membrane content of RANKL (126). Picherit et al. demonstrated that in ovariectomised rat model of postmenopausal osteoporosis, oral administration of daidzein arrested both cancellous and cortical bone loss or only cortical bone loss, while manifesting no estrogenic activity on the uterus. Therefore, this proves that daidzein has no adverse effects on the uterus (127). Treatment with daidzein on ovariectomized mice significantly reduced the production of ROS and TNF-a by activated T-cells, both of which are involved in the stimulation of osteoclastogenesis (128). In osteoblast like MG-63 cells, administration of daidzein caused a remarkable elevation in the levels of ALP and COL-1 and also protected against cisplatin induced apoptosis *via* an ER-dependent MEK/ERK and PI3K/AKT activation (129). Daidzein promoted osteoblast proliferation and differentiation *via* the BMP pathway, which upregulated the phosphorylated levels of SMAD 1/5/8. This in turn, led to an increase in the expression of osteogenic marker genes, including ALP, RUN-X2, COL-1 and OSX (130). Furthermore, daidzein demonstrated anti-osteoclastic activity in RAW264.7 cells by downregulating the expression levels of TNF-a induced c-Fos and NFAT-c1 (both of which are important regulators of osteoclast differentiation) in an ER dependent manner. In addition, daidzein inhibited nuclear translocation of NFAT-c1 and also reduced the levels of NF-kB and DC-STAMP levels (131). However, high levels of daidzein cannot not always be beneficial. A correct balance is always required for optimum activity. A study by Dang et al., using mouse bone marrow cells and mouse osteoprogenitor KS483 cells has shown that at concentrations below 20 μM, they inhibit osteogenesis and at concentrations higher than 30 μM, it stimulates adipogenesis (124). This proves that a proper amount of daidzein should be taken, and high or lower levels may not tend to be beneficial to the human body.

#### 5.3.7 Luteolin

Luteolin is a flavonoid found in many herbal extracts and has been a part of the traditional culture in Asian countries through medicines and supplements. Exposure of mouse bone marrow derived macrophages (BMMs) to luteolin inhibited osteoclast differentiation induced by RANKL and also downregulated the expression of osteoclast related genes such as NFATc1, c-Src, DC-STAMP, MMP-9, CTSK and TRAP. Moreover, luteolin suppressed bone resorption in a dose-dependent manner in mature osteoclasts incubated with RANKL and M-CSF (132). In RAW264.7 cells, luteolin inhibited the formation of mature TRAP-positive osteoclasts induced by RANKL *via* the suppression of activating transcription factor (ATF2) downstream of p38 MAPK and NFATc1, thus inhibiting bone resorption. This was accompanied by the disruption of actin rings of the osteoclasts (133). The effects of luteolin on the prevention of bone loss in experimental periodontitis in Wistar rats were assessed and it was found that treatment with luteolin remarkably decreased the alveolar bone loss by attenuating osteoclastogenic activity and production of osteoclastogenic markers including MMP-9 and RANKL. Besides, it upregulated osteoblastic activity *via* the increased expression of osteogenic markers such as tissue inhibitor of metalloproteinase (TIMP-1), BMP-2, and OPG expressions (134). Nash et al. demonstrated that Luteolin-treated mouse osteoblasts exhibited elevated ALP activity and collagen formation *via* interactions with estrogen receptors (135). Luteolin treatment of MC3T3-E1 osteoblasts abrogated the 3-morpholinosydnonimine (SIN-1)-induced production of oxidative stress markers which included NO, PGE2, TNF-a and IL-6, thus preventing osteoclastogenesis and bone resorption in diseases linked with the overproduction of inflammatory mediators such as arthritis (136). In cultured human periodontal ligament cells (HPDLCs), administration of 1µmol of luteolin strongly enhanced cell viability, ALP activity and increased calcified nodules content. Additionally luteolin significantly upregulated the mRNA and protein expression levels of osteoblast specific markers such as ALP, BMP2, OSX and OCN and the relative expression levels of β-catenin and cyclin D1 (137). Yang et al., demonstrated that in murine calvarial osteoblasts administration of luteolin suppressed the IL-1b-induced expressions of MMP-9 and MMP-13 *via* a possible inhibition of the ERK pathway, thus preventing excessive degradation of bone matrix (138). Luteolin dose dependently suppressed the mRNA and protein expression levels of pro-inflammatory cytokines and mediators including TNF-a, IL-6, COX-2 and iNOS in LPS-stimulated mouse alveolar macrophage MH-S and peripheral macrophage RAW 264.7 cell lines *via* inhibition of phosphorylated NF-kB and AP-1 mediated through blockage of Akt and IkB kinase (IKK) phosphorylation. Further, luteolin inhibited the production of ROS as well (139). In a study by Abasi et al., it was found that luteolin at lower concentrations conferred protection against high-glucose-induced cell death compared to its cytotoxic effects at high doses. Thus, in order to utilise the protective cations of luteolin, it is safest to avoid consuming high doses of luteolin in food supplements (140).

#### 5.3.8 Genistein

**Figure 6:**
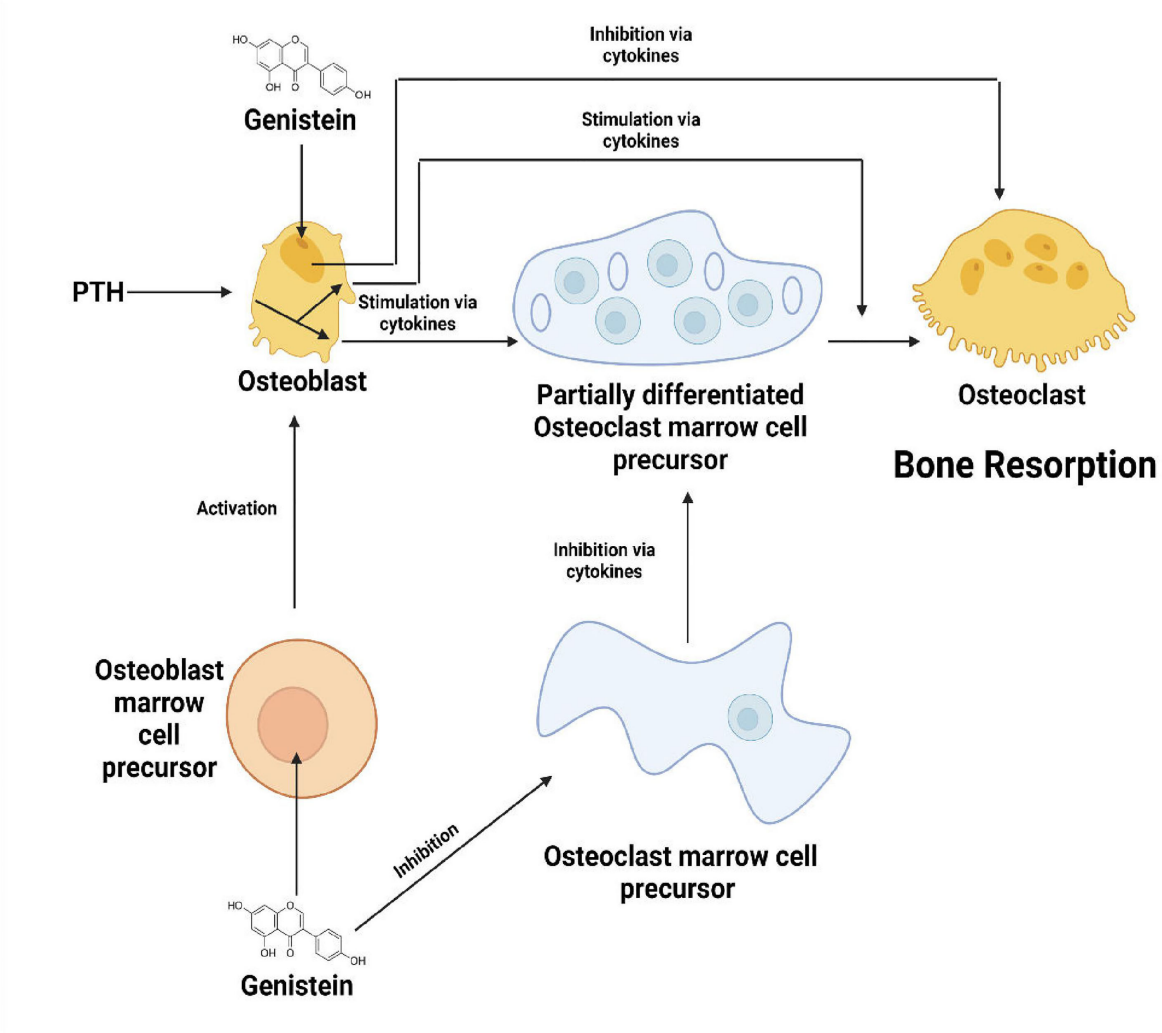
Effects of genistein on osteoblasts, osteoclasts and their precursor cells.

Genistein, a phytoestrogen, is a non-steroidal compound, that shows structural similarity to estradiol-17β. This enables genistein to bind to sex hormone binding proteins and estrogen receptors, thus exhibiting anti-estrogenic and estrogenic properties, the former being done by competing with estradiol with estrogen receptors (141, 142). Anderson et al. discovered a tendency in OVX rats treated with genistein to maintain a better bone mass when compared to the untreated control rats and conjugated estrogen-treated rats, with the low-dose genistein treated groups exhibiting the highest numerical effect on bone retention. Several studies have implied that at low doses genistein acts through estrogen receptors, thus rendering bone-preserving effects. However, it has also been shown that genistein at high doses might induce multiple cellular effects and may not necessarily cause estrogen receptor activation. Thus, further studies are required to determine the effects of non-pharmacological doses of genistein (143). In a study conducted by Li et al. on Sprague Dawley rats, it was found that genistein at both high and low doses, caused a remarkable increase in the BMD, bone volume and also resulted in denser subchondral trabecular bone *in vivo*. At low doses, genistein upregulated the mRNA expression levels of osteogenic markers including ALP, OCN, OPG, ERα and ERβ, whereas it downregulated the osteoclastogenic marker RANKL both *in vivo* and invitro. High dose genistein decreased the mRNA levels of bone homeostasis related markers such as ALP, OCN, OPG, RANKL and ERα, while it increased ERβ expression levels invitro and *in vivo*, thus not only inhibiting bone resorption but also bone formation at higher doses- (144). Fanti et al., demonstrated that treatment with genistein of OVX rats lead to an approximate 50% percent reduction in distal femur cancellous bone loss and loss of whole tibia BMD. Highest genistein dose (25 mg/g/day) resulted in larger uterine size compared to the intermediate dose which provided maximum bone-sparing effects but lesser uterine size, thus suggesting a possible non-estrogen mediated mechanism of genistein such as direct interaction with cellular enzymes including *via* direct interaction with cellular enzymes as diverse as 5-LOX, COX, cyclic AMP phosphodiesterase, protein kinases, DNA topoisomerase II and 11b-hydroxysteroid dehydrogenase (**Figure 6**). Moreover, genistein treatment suppressed the elevated levels of pro-inflammatory cytokine TNF-a, an inhibitor of osteogenesis (145). In MC3T3 pre-osteoblastic cells, treatment with genistein altered the expression levels of genes associated with cell proliferation, cell migration, cell differentiation, and inflammatory responses. Successive knockdown analyses showed that two upregulated genes (Ereg and Efcab2) and three downregulated genes (Hrc, Gli1, and Iftm5) play crucial roles in the differentiation of osteoblasts *via* increasing the expressions of osteoblast-associated markers such as RUNX2, ALP and BMP-2 (146). Administration of genistein to human bone marrow stromal cells suppressed its differentiation into adipocytes by inhibiting the mRNA levels of PPARg and CCAAT/enhancer binding proteins (C/EBPs), while it enhanced osteoblastogenesis, thus preventing bone loss associated with excessive adipogenesis (147). Besides, genistein was also found to increase the expression levels of b-catenin and reduced the levels of IL-6 in Sprague Dawley rats (148). Liao et al. have shown that genistein promotes osteoblastic differentiation by the activation of p38 MAPK-RUNX2 pathway. Moreover, several other studies have revealed a possible cross talk between this pathway and other pathways mediated by BMP and protein kinase C (PKC) (149). Genistein also has shown to induce osteoblast proliferation and differentiation from BMSCs through the involvement of ER–NO–cGMP pathway (150). The expressions of two main osteoclastogenic markers c-Fos and NFATc1, were found to be inhibited by genistein. Furthermore, genistein inhibited RANKL-induced degradation of IkB and nuclear translocation of NF-kB and also suppressed the expressions of IL-1 and CTSK mediated by tyrosine kinase-NF-kB pathway. These effects led to the inhibition of differentiation of osteoclasts and subsequent bone resorption (89).

#### 5.3.9 Hesperidin

**Figure 7:**
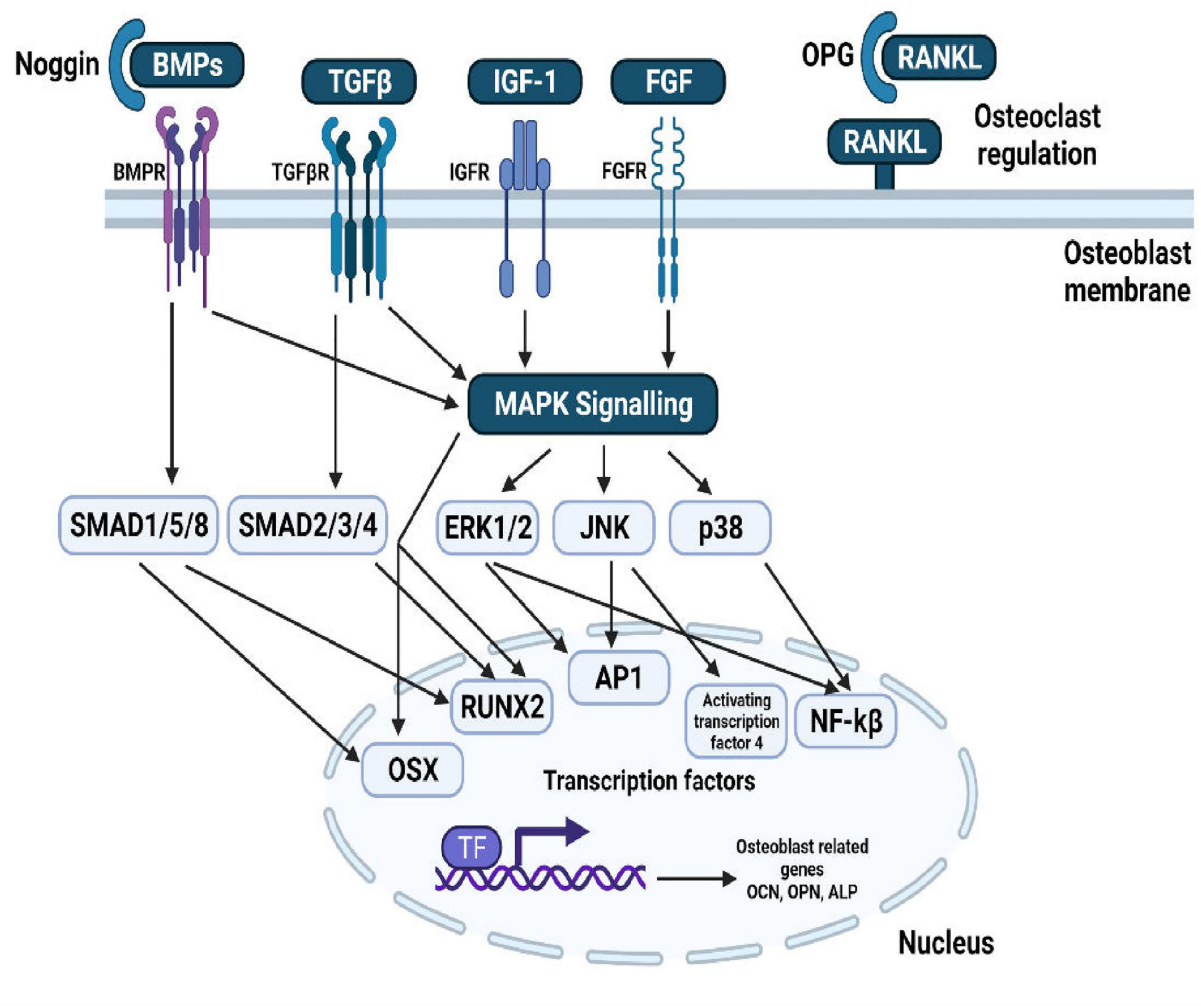
Effect of hesperidin on signalling pathway regulating differentiation of osteoblasts.

Hesperidin, also called Hesperetin-7-O-glucuronide is a flavonoid abundantly found in citrus fruits and belongs to the flavonoid subgroup called flavonones. Hesperidin is a glycoside flavonoid which when absorbed gets hydrolysed into the aglycone form by gut microbiota and undergoes further metabolic changes-1 (151). Several studies have reported hesperidin to act as a potential bioactive compound in maintaining bone health in OVX rat models (152). In primary osteoblasts obtained from wistar rats, hesperidin, was found to upregulate the mRNA levels of ALP and OCN *via* upregulation of RUNX2 and OSX, the two important transcription factors in relation to osteoblasts, which are a part of the MAPK and BMP signalling pathways (**Figure 7**). Phosphorylation of SMAD1/5/8 complex also seemed to be increased, thus suggesting the participation of the BMP pathway through activation of SMAD1/5/8. Moreover, noggin, a protein secreted by osteoblasts and known to hinder the BMP pathway was found to be downregulated by hesperidin (151). Besides, treatment with hesperidin showed slight modulation in the levels of c-Jun and c-Fos, which form a part of the transcription factor AP-1 responsible for the activation of osteoblast-related genes. This indicates the possible intervention of hesperidin through the MAPK signalling pathways (152). In periodontal ligament stem cells (PDLSCs), administration of hesperitin, increased the mRNA level of the osteogenic transcription factor Fos-related antigen-1 (FRA-1) and also the protein levels of OPN and COL-1A. Under conditions of high glucose, the ROS produced by PDLSCs were scavenged by hesperitin. Furthermore, hesperitin also stimulated the activation of Wnt/b-catenin pathway mediated by the activation of PI3K/AKT signalling (153). A study by Kim et al., demonstrated a possible antiresorptive effect of hesperitin through the inhibition of four pathways namely NIK/IKK, ERK, p38, and JNK, which in turn suppressed the NF-kB signalling responsible for osteoclastogenesis and also showed effects on the redox regulating transcription factors Trx/Ref-1 (154). Additionally, exposure to hesperidin of male gonad-intact senescent rats, attenuated the production of the pro-inflammatory cytokine IL-6 (155). Although the exact mechanism of action of hesperidin hasn’t been elucidated, the above-mentioned pathways have been discovered as of yet to be regulated by hesperidin.

#### 5.3.10 Apigenin

**Figure 8:**
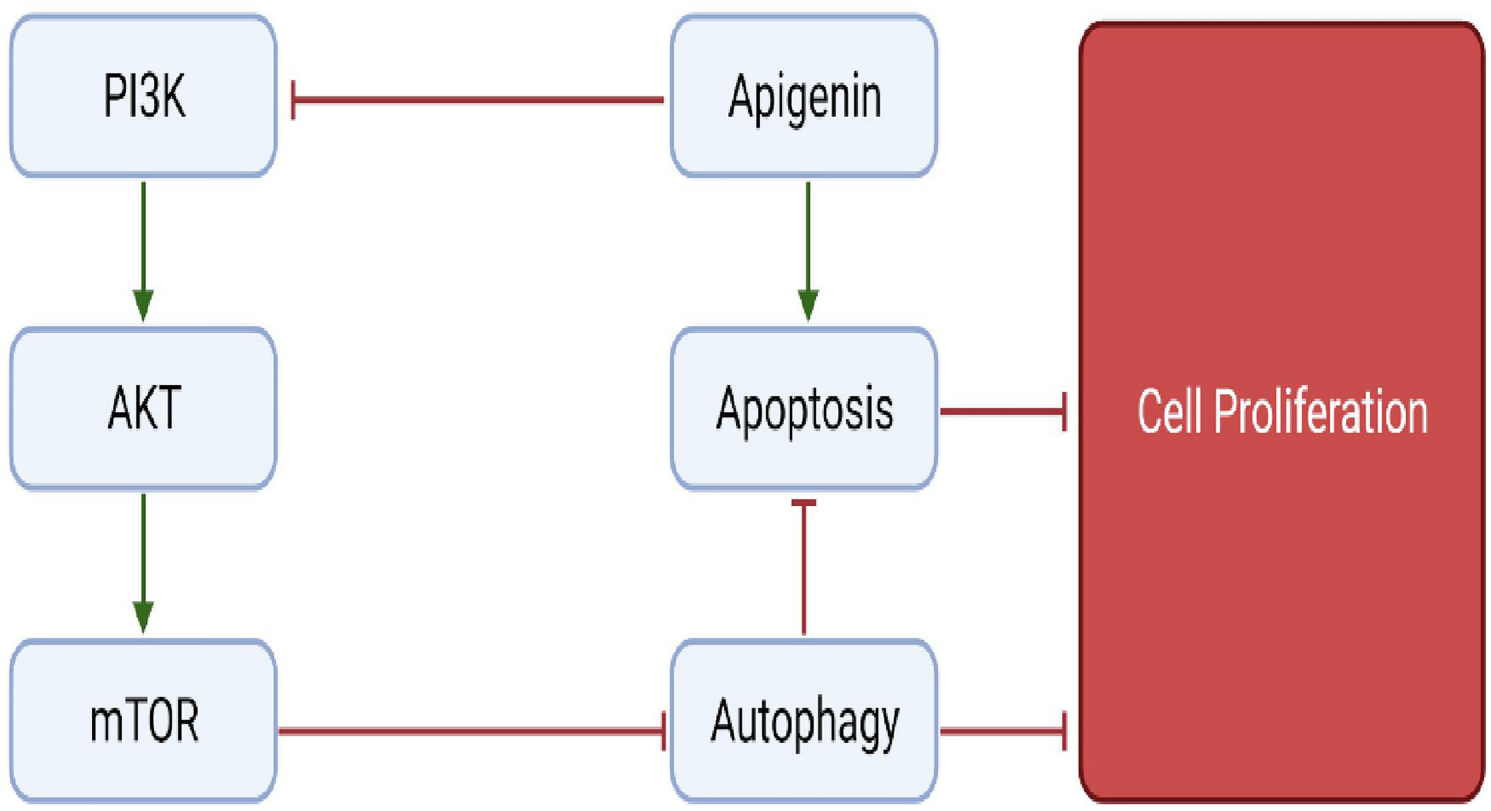
Regulation of autophagy and apoptotic pathways by apigenin.

**Figure 9:**
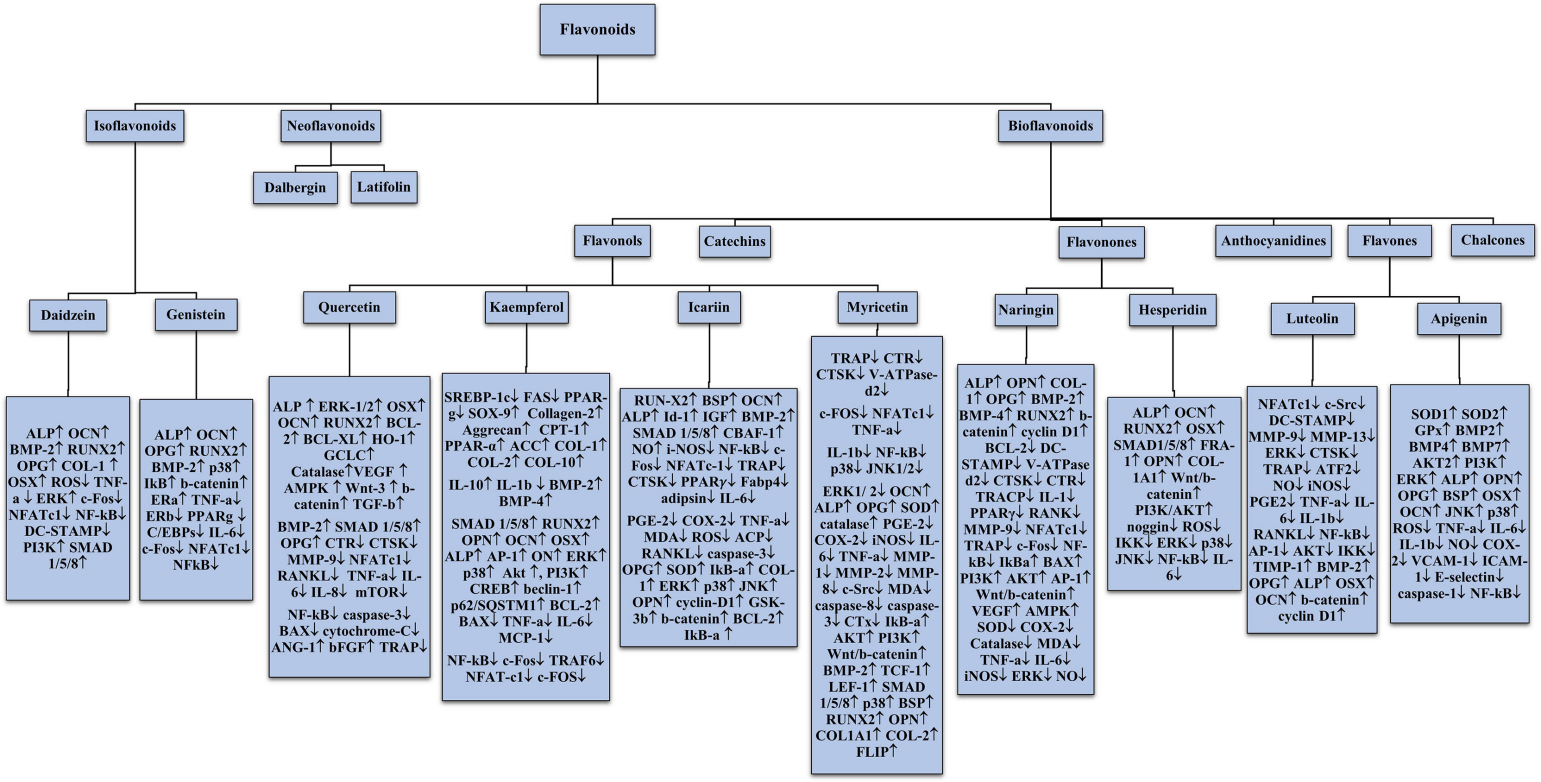
Overall schematic depiction of flavonoids regulating molecules in bone remodelling.

Apigenin is a flavonoid belonging to the subgroup flavone and is widely present in several fruits and vegetables such as olives, apples and parsley. Although only minimum information is present on the role of apigenin in bone metabolism, a few studies indicate the role of apigenin in preventing bone loss (156). Pre-treatment of H2O2 induced MC3T3-E1 cells with apigenin, caused an upregulation of anti-oxidant enzymes SOD1, SOD2 and glutathione peroxidase (GPx), thus counteracting the ROS produced. Further, apigenin remarkably increased the expression levels of genes responsible for osteoblast differentiation such as ALP, OPN, OPG, BSP, OSX, OCN and BMPs (BMP2, BMP4 and BMP7). Other anti-oxidant properties of apigenin include activating H2O2-induced reduced expression of AKT2, PI3K and ERK, all of which are key-regulators of pathways involved in survival, thus inhibiting apoptosis osteoblasts (**Figure 8**). These findings suggest the role of apigenin in the treatment of bone diseases associated with oxidative stress (157). Apigenin treatment of TNF-a-induced MC3T3-E1 osteoblasts, reduced its production of IL-6 and NO involved in bone resorption, suggesting apigenin’s intervention in treating bone disorders such as osteoporosis characterised by excessive production of inflammatory cytokines (158). In a study by Lee et al., it was demonstrated that apigenin supressed the activity of collagenase in RA and also showed that apigenin inhibited LPS-induced production of NO and COX-2 by RAW 264.7 macrophage cells. In addition, apigenin significantly attenuated the TNFa-induced adhesion of monocytes to human umbilical vein endothelial cell (HUVEC) monolayer and TNF-a-stimulated elevation of vascular cellular adhesion molecule-1 (VCAM-1), intracellular adhesion molecule-1 (ICAM-1), and E-selectin-mRNA, all of which are involved in RA (159). Treating LPS-induced macrophages with apigenin, profoundly suppressed the production of IL-6, IL-1β, and TNF-α *via* regulating various signalling pathways. Apigenin suppressed LPS-induced production of IL-1β by disrupting caspase-1 activation *via* hampering the inflammasome assembly. Also, apigenin arrested the LPS-stimulated production of IL-6 and IL-1β by decreasing the mRNA stability through inhibition of ERK1/2 activation. Additionally, apigenin inhibited the activation of NF-kB *via* induced by TNF-α and IL-1β thus providing evidence to use apigenin for potentially treating inflammatory bone diseases (160). Furthermore, Zhang et al., have shown the involvement of JNK and p38 MAPK signalling pathways in stimulating osteoblast differentiation *via* upregulation of osteoblast-specific genes (161). In conclusion, the pathways discussed above provide evidence to use apigenin as a possible intervention in the treatment of bone-related diseases. **Figure 9** depicts an overview of the flavonoids that regulate molecular mechanism in bone remodelling.

#### 5.3.11 Other Flavonoid

Puerarin, a natural isoflavone isolated from the Chinese herb Pueraria lobata, exhibits osteogenic effects similar to 17-β-estradiol, suggesting a therapeutic role in the treatment of osteoporosis in the future. Puerarin treatment on rat osteoblasts increased the levels of ALP and stimulated osteoblastic proliferation *via* a possible mediation of the PI3K/Akt pathway (162). Puerarin alleviated pathological bone graft defects and apoptosis of BMSCs and increased their proliferation and differentiation. Further, it decreased the levels of proinflammatory cytokines and promoted the levels of anti-inflammatory cytokines, thus ameliorating bone loss *via* inflammation (163). In human osteoblasts (hOBs), treatment with puerarin was shown to inhibit serum-free-induced apoptosis by upregulating the expression of BCL-2 and downregulating the expression of BAX through the activation ERK-1/2 signalling pathway (164). Besides puerarin is well accepted as an autophagy regulator and osteoclastogenesis inhibitor, with the exact role of autophagy in puerarin-regulated osteoclastogenesis still being unclear (165). In RANKL-induced BMMs, osteoclastogenesis was alleviated with puerarin treatment, which inhibited the expression of osteoclastogenic genes and the TRAF6/ROS-dependent MAPK/NF-κB signalling pathway (166). Petunidin, a compound belonging to the flavonoid family anthocyanin has been shown to be a promising natural agent in inhibiting osteoclastogenesis and promoting bone formation. Treating RAW264.7 cells with petunidin significantly inhibited osteoclastogenesis by suppressing the mRNA expression of osteoclastogenic markers c-Fos, NFATc1, MMP9, CTSK, and DC-STAMP. Petunidin stimulated the gene expression of osteogenic markers BMP-2 and OCN, whereas it inhibited mRNA expression of MMP-2, MMP-9, MMP-13 and the proteolytic activities of MMP-9 and MMP-13 in MC3T3-E1 cells (167). The isoflavonoid formononetin has been suggested to be a natural selective estrogen receptor modulator (SERM), and exhibit estrogenic activity on bone cells, thus inhibiting the development of osteoporosis in post-menopausal women (168). A study by Singh et al. revealed that treatment with formononetin on overiectomised (OVx) osteopenic mice repaired the cortical bone defect and promoted bone regeneration accompanied by elevated expression of osteogenic markers BMP-2, RUNX2 and OCN (169). Formononetin treatment on C2C12 progenitor cells remarkably enhanced ALP activity, calcium deposition, and the expression of osteogenesis specific markers including ALP, RUNX2, OCN and BMP isoforms. It was also demonstrated that osteogenic differentiation in these cells treated with formononetin was enhanced by p38 MAPK dependent SMAD 1/5/8 signalling pathways (170). In BMMs, treatment with formononetin regulated OPG and RANKL expression levels, and inhibited RANKL induced TNF-α, IL-1β, IL-6, MCP-1 and macrophage inflammatory protein-1α (MIP-1α). These were accompanied by a reduction in RANKL induced activation of the NF-κB p65 subunit, degradation of IκBα, activation of AKT, ERK, JNK and p38 MAPK (171). In addition, formononetin inhibited classic osteoclastogenic markers significantly. Furthermore, it possesses angiogenic properties required for bone fracture healing and upregulates expression of VEGF and VEGF-R2 (172). Naringenin a dihydro flavonoid compound abundantly found in fruits such as orange, pomelo and drynaria has shown to possess osteogenic effects. In BMSCs, treatment with naringenin upregulated the gene and protein expression levels of ALP, RUNX2, C-X-C chemokine receptor type 4 (CXCR4) and stromal cell-derived factor 1 (SDF-1) *via* the SDF-1/CXCR4 signalling pathway (173). Calycosin, an isoflavonoid phytoestrogen, significantly suppressed osteoclast formation from BMMs and inhibited the expression of osteoclastogenic markers, including CTSK, TRAP and MMP-9. Moreover, calycosin attenuated the expression levels of NFATc1 and c-Fos *via* inhibition of activation of NF-kB and MAPKs thereby preventing bone resorption (174). Curcuma longa, a member of the family Zingiberaceae commonly referred to as turmeric, contains an important flavonoid named curcumin. A study by Folwarczna et al. exhibited that curcumin reduced serum estradiol and mineralization, and increased bone formation and histomorphometric properties of the bone (175–178). Curcumin was found to downregulate the Wnt/β-catenin pathway, AKT pathway, BCL-2, NF-kB, COX-2 and activated GSK-3β, thus preventing oxidative stress and inflammatory responses induced by these pathways (177, 179–187). A study by Notoya et al., utilising rat calvarial osteoblast-like cells, showed that curcumin inhibited the proliferation of osteoblasts without induction of apoptosis. This occurred due to the expression of p21 protein, which resulted in the arrest of cell cycle (188). In another study by Yamaguchi et al. with an analogue of curcumin UBS109, it was found to increase SMAD activity, BMP-induced SMAD activation and TGF-β-induced SMAD activation. It was also found to inhibit TNF-α-induced SMAD suppression. This might be crucial to enhance the differentiation of osteoblasts (189). Also, curcumin slightly inhibited the enhancement of RANKL by IL-1α in human bone marrow stromal cells (190). Epigallocatechin-3-gallate is a flavonoid found abundantly in green tea. Epigallocatechin (EGCG) was found to have promote differentiation of osteoblasts in murine BMSCs. In a study by Lin et al., EGCG showed upregulation of osteogenic-related genes including osteocalcin, RUNX2, OCN, ALP and BMP2, resulting in increased mineralization in a cultured mesenchymal stem cell line derived from bone marrow (191). The expression of RUNX2 and OSX, which are important for mesenchymal stromal cells to differentiate to osteoblasts, was increased by EGCG, and thereby resulted in increased osteogenesis (192). Oleuropein, a flavonoid found in green olives and the olive tree, has recently been deeply researched for its multiple health benefits (193). In a study by Santiago-Mora et al. using the periodontitis model in rats, Oleuropein downregulated the genes linked with adipogenesis such as lipoprotein lipase and PPAR‐γ and upregulated the factors promoting osteogenesis such as OCN, RUNX2 and ALP and eventually enhanced osteoblastic differentiation (194). Moreover, oleuropein reduced JNK, p38 MAPK and ERK1/2, and prevented the translocation of NF‐KB from cytosol to nucleus which is important for the activation of NF‐KB (195). Castejon et al. also demonstrated a downregulation of MAPK and NF‐KB pathway, reduced MMP‐3, COX‐2, TNF‐α, MMP‐1 and IL‐6 levels in IL‐1β-induced synovial fibroblast cells by oleuropein (196, 197). OCN and BMP4 are augmented by this flavonoid, and TRAP osteoclasts are inhibited (194, 198). BMP4 is linked with high OPG production, and this leads to a higher rate of osteoblastogenesis (199).

## 6 Conclusion and Future Perspective

Bone-related disorders as such are a growing problem in aging populations especially post-menopausal women experiencing acute estrogen deficiency. The long-term progression of these diseases give rise to serious consequences such as fractures which create significant negative impacts including reduced quality of life, sustained disability and a growing economic burden due to their high medical costs. The current treatment options consisting of antiresorptive agents (such as bisphosphonates, hormone-replacement therapy, selective oestrogen-receptor modulators and anti-RANKL antibodies) and/or anabolic agents (such as intermittent low doses of teriparatide and antisclerostin antibodies) are not free from adverse effects that limit their use (66). This is where flavonoids come into role. These naturally derived phytochemicals possessing potent bone conserving properties beyond calcium and vitamin D exhibit fewer or no side effects compared to conventional therapies. A number of flavonoids are being evaluated for their properties beyond their chemical anti-oxidant capacity, such as anti-inflammatory effects. By regulating cell signalling pathways that influence osteoblast and osteoclast differentiation, these bioactive compounds have been reported to promote bone formation and inhibit bone resorption. However, there is no single mechanism that can elucidate the actions of flavonoids, rather it is a combination of a myriad of pathways. Despite the presence of several gaps, attempts are being made to develop a unifying model to integrate the identified molecular targets and signalling pathways and show how flavonoids from different plant sources might affect them (8). Only a small number of studies on flavonoids have been extrapolated to human clinical trials. In a double-blind placebo randomised controlled study by Hassan et al., on type 2 diabetes mellitus patients, the effects of quercetin administration on biomarkers of bone mineralisation were investigated. It was found that patients who received an oral supplementation of quercetin at 500mg/day for a period of 3 months exhibited increased levels of serum OCN, Vitamin D and calcium compared to their pre-treatment levels (66). A similar study carried out for a combined dosage administration of icariin, genistein and daidzein for 24 months in postmenopausal women showed reduced bone loss and improved BMD in the lumbar spine and femoral neck (90). Furthermore, ongoing studies suggest the possibility of incorporation of flavonoids in bone scaffolds and grafts to ensure local administration and sustained release of flavonoids which can aid in quicker bone healing. This strategy has been considered to overcome the shortcomings concerned with bioavailability, stability and other biopharmaceutical properties of flavonoids so that a desired concentration can be maintained at the target site (200). Despite having such tremendous implications on bone health, only a limited number of studies on flavonoids have been extended beyond animal models. In order to translate these animal data to dietary interventions in humans, we also require comparative data of the various sources of flavonoids. Therefore, proper identification of the flavonoids’ sources, bioactive ingredients and their effective doses remains crucial to undertake and invest in future clinical trials (8). However, the study of interactions of flavonoids with various cellular pathways and their potential to aid in the prevention or repair of bone defects possesses tremendous scope and is definitely a rich area for future research.

## Author Contributions

PR, RJ, SS, and SV collected literature and drafted the manuscript. AD provided technical help. SV secured funding, designed the work, and approved the final submitted manuscript. All authors contributed to the article and approved the submitted version.

## Funding

This work was supported by Department of Science and Technology, INSPIRE Faculty Program, Government of India for the research grant to SV (grant no. DST/INSPIRE/04/2017/002913).

## Conflict of Interest

The authors declare that the research was conducted in the absence of any commercial or financial relationships that could be construed as a potential conflict of interest.

## Publisher’s Note

All claims expressed in this article are solely those of the authors and do not necessarily represent those of their affiliated organizations, or those of the publisher, the editors and the reviewers. Any product that may be evaluated in this article, or claim that may be made by its manufacturer, is not guaranteed or endorsed by the publisher.

## References

[1] Raggatt LJ Partridge NC. Cellular and Molecular Mechanisms of Bone Remodeling. J Biol Chem (2010) 285:25103–8. doi: 10.1074/jbc.R109.041087 PMC2919071 20501658

[2] Vimalraj S. Alkaline Phosphatase: Structure, Expression and Its Function in Bone Mineralization. Gene (2020) 754:144855. doi: 10.1016/j.gene.2020.144855 32522695

[3] Kular J Tickner J Chim SM Xu J. An Overview of the Regulation of Bone Remodelling at the Cellular Level. Clin Biochem (2012) 45:863–73. doi: 10.1016/j.clinbiochem.2012.03.021 22465238

[4] Kenkre JS Bassett JHD. The Bone Remodelling Cycle. Ann Clin Biochem (2018) 55:308–27. doi: 10.1177/0004563218759371 29368538

[5] Kumar S Pandey AK. Chemistry and Biological Activities of Flavonoids: An Overview. Sci World J (2013) 2013:162750. doi: 10.1155/2013/162750 PMC3891543 24470791

[6] Panche AN Diwan AD Chandra SR. Flavonoids: An Overview. J Nutr Sci (2016) 5:e47. doi: 10.1017/jns.2016.41 28620474 PMC5465813

[7] Bellavia D Dimarco E Costa V Carina V De Luca A Raimondi L. Flavonoids in Bone Erosive Diseases: Perspectives in Osteoporosis Treatment. Trends Endocrinol Metab (2021) 32:76–94. doi: 10.1016/j.tem.2020.11.007 33288387

[8] Weaver CM Alekel DL Ward WE Ronis MJ. Flavonoid Intake and Bone Health. J Nutr Gerontol Geriatr (2012) 31:239–53. doi: 10.1080/21551197.2012.698220 PMC3898177 22888840

[9] Vimalraj S Saravanan S Subramanian R. Rutin-Zn(II) Complex Promotes Bone Formation - A Concise Assessment In Human Dental Pulp Stem Cells and Zebra. Chem Biol Interact (2021) 349:109674. doi: 10.1016/j.cbi.2021.109674 34562440

[10] Hasan Waheed Janabi A Ali Kamboh A Saeed M Xiaoyu L BiBi J Majeed F. Spectroscopic Investigation on the Interaction of DNA With Superparamagnetic Iron Oxide Nanoparticles Doped With Chromene *via* Dopamine as Cross Linker. Iran J Basic Med Sci (2020) 23:140–53. doi: 10.22038/IJBMS.2019.35125.8353 PMC7211351 32405356

[11] Mukwaya E Xu F Wong MS Zhang Y. Chinese Herbal Medicine for Bone Health. Pharm Biol (2014) 52:1223–8. doi: 10.3109/13880209.2014.884606 24963946

[12] Li J Bai L Li X He L Zheng Y Lu H. Antidiabetic Potential of Flavonoids From Traditional Chinese Medicine: A Review. Am J Chin Med (2019) 47:933–57. doi: 10.1142/S0192415X19500496 31248265

[13] Liu HX Lin WH Wang XL Yang JS. Flavonoids From Preparation of Traditional Chinese Medicines Named Sini-Tang. J Asian Natural Prod Res (2005) 7:139–43. doi: 10.1080/1028602042000204063 15621616

[14] Shi Z Li T Liu Y Cai T Yao W Jiang J. Hepatoprotective and Anti-Oxidative Effects of Total Flavonoids From Qu Zhi Qiao (Fruit of Citrus Paradisi Cv.Changshanhuyou) on Nonalcoholic Steatohepatitis *In Vivo* and *In Vitro* Through Nrf2-ARE Signaling Pathway. Front Pharmacol (2020) 11. doi: 10.3389/fphar.2020.00483 PMC7189874 32390839

[15] Feng X McDonald JM. Disorders of Bone Remodeling. Annu Rev Pathol (2011) 6:121. doi: 10.1146/annurev-pathol-011110-130203 20936937 PMC3571087

[16] Rucci N. Molecular Biology of Bone Remodelling. Clin Cases Miner Bone Metab (2008) 5(1):49–56. 22460846 PMC2781193

[17] Parra-Torres AY Valdés-Flores M Orozco L Velázquez-Cruz R. Molecular Aspects of Bone Remodeling. In: Topics in Osteoporosis (2013) Mexico: InTechOpen. doi: 10.5772/54905

[18] Hayman AR. Tartrate-Resistant Acid Phosphatase (TRAP) and the Osteoclast/Immune Cell Dichotomy. Autoimmunity (2008) 41:218–23. doi: 10.1080/08916930701694667 18365835

[19] Hsiao C-YY Chen T-HH Chu T-HH Ting Y-NN Tsai P-JJ Shyu J-FF. Calcitonin Induces Bone Formation by Increasing Expression of Wnt10b in Osteoclasts in Ovariectomy-Induced Osteoporotic Rats. Front Endocrinol (2020) 11:613. doi: 10.3389/fendo.2020.00613 PMC7506163 33013696

[20] Teitelbaum SL. Osteoclasts: What do They do and How do They do It? Am J Pathol (2007) 170:427–35. doi: 10.2353/ajpath.2007.060834 PMC1851862 17255310

[21] Boyce B Yao Z Xing L. Osteoclasts Have Multiple Roles in Bone in Addition to Bone Resorption. Crit Rev Eukaryot Gene Expr (2009) 19:171–80. doi: 10.1615/CritRevEukarGeneExpr.v19.i3.10 PMC2856465 19883363

[22] Hofbauer LC Heufelder AE. Role of Receptor Activator of Nuclear Factor-κb Ligand and Osteoprotegerin in Bone Cell Biology. J Mol Med (2001) 79:243–53. doi: 10.1007/s001090100226 11485016

[23] Horwood NJ Elliott J Martin TJ Gillespie MT. Osteotropic Agents Regulate the Expression of Osteoclast Differentiation Factor and Osteoprotegerin in Osteoblastic Stromal Cells. Endocrinology (1998) 139:4743–6. doi: 10.1210/endo.139.11.6433 9794488

[24] Kim N Odgren PR Kim DK Marks SC Choi Y. Diverse Roles of the Tumor Necrosis Factor Family Member TRANCE in Skeletal Physiology Revealed by TRANCE Deficiency and Partial Rescue by a Lymphocyte-Expressed TRANCE Transgene. Proc Natl Acad Sci U S A (2000) 97:10905–10. doi: 10.1073/pnas.200294797 PMC27122 10984520

[25] Lee SE Woo KM Kim SY Kim HM Kwack K Lee ZH. The Phosphatidylinositol 3-Kinase, P38, and Extracellular Signal-Regulated Kinase Pathways are Involved in Osteoclast Differentiation. Bone (2002) 30:71–7. doi: 10.1016/S8756-3282(01)00657-3 11792567

[26] Nakchbandi IA Grey A Masiukiewicz U Mitnick M Insogna K. Cytokines in Primary Hyperparathyroidism. Parathyroids (2001) 81:411–21. doi: 10.1016/B978-012098651-4/50027-4

[27] Boyce BF Yoneda T Lowe C Soriano P Mundy GR. Requirement of Pp60c-Src Expression for Osteoclasts to Form Ruffled Borders and Resorb Bone in Mice. J Clin Invest (1992) 90:1622–7. doi: 10.1172/JCI116032 PMC443211 1383278

[28] Grigoriadis AE Wang ZQ Cecchini MG Hofstetter W Felix R Fleisch HA. C-Fos: A Key Regulator of Osteoclast-Macrophage Lineage Determination and Bone Remodeling. Science (1994) 266:443–8. doi: 10.1126/science.7939685 7939685

[29] Roodman GD Windle JJ. Paget Disease of Bone. J Clin Invest (2005) 115:200–8. doi: 10.1172/JCI24281 PMC546434 15690073

[30] Takayanagi H. Mechanistic Insight Into Osteoclast Differentiation in Osteoimmunology. J Mol Med (Berlin Germany) (2005) 83:170–9. doi: 10.1007/s00109-004-0612-6 15776286

[31] Komori T Yagi H Nomura S Yamaguchi A Sasaki K Deguchi K. Targeted Disruption of Cbfa1 Results in a Complete Lack of Bone Formation Owing to Maturational Arrest of Osteoblasts. Cell (1997) 89:755–64. doi: 10.1016/S0092-8674(00)80258-5 9182763

[32] Lian JB Stein GS Javed A van Wijnen AJ Stein JL Montecino M. Networks and Hubs for the Transcriptional Control of Osteoblastogenesis. Rev Endocr Metab Disord (2006) 7:1–16. doi: 10.1007/s11154-006-9001-5 17051438

[33] Nakashima K Zhou X Kunkel G Zhang Z Deng JM Behringer RR. The Novel Zinc Finger-Containing Transcription Factor Osterix is Required for Osteoblast Differentiation and Bone Formation. Cell (2002) 108:17–29. doi: 10.1016/S0092-8674(01)00622-5 11792318

[34] Qin L Qiu P Wang L Li X Swarthout JT Soteropoulos P. Gene Expression Profiles and Transcription Factors Involved in Parathyroid Hormone Signaling in Osteoblasts Revealed by Microarray and Bioinformatics. J Biol Chem (2003) 278:19723–31. doi: 10.1074/jbc.M212226200 12644456

[35] Westendorf JJ Kahler RA Schroeder TM. Wnt Signaling in Osteoblasts and Bone Diseases. Gene (2004) 341:19–39. doi: 10.1016/j.gene.2004.06.044 15474285

[36] Murshed M Harmey D Millán JL McKee MD Karsenty G. Unique Coexpression in Osteoblasts of Broadly Expressed Genes Accounts for the Spatial Restriction of ECM Mineralization to Bone. Genes Dev (2005) 19:1093–104. doi: 10.1101/gad.1276205 PMC1091743 15833911

[37] Eriksen EF. Cellular Mechanisms of Bone Remodeling. Rev Endocr Metab Disord (2010) 11:219–27. doi: 10.1007/s11154-010-9153-1 PMC3028072 21188536

[38] Sozen T Ozisik L Calik Basaran N. An Overview and Management of Osteoporosis. Eur J Rheumatol (2017) 4:46–56. doi: 10.5152/eurjrheum.2016.048 28293453 PMC5335887

[39] Raisz LG. Pathogenesis of Osteoporosis: Concepts, Conflicts, and Prospects. J Clin Invest (2005) 115:3318–25. doi: 10.1172/JCI27071 PMC1297264 16322775

[40] Manolagas SC. Cellular and Molecular Mechanisms of Osteoporosis. Aging Clin Exp Res 1998 (2013) 10(3):182–90. doi: 10.1007/BF03339652 9801728

[41] Flemmig TF. Periodontitis. Ann Periodontol (1999) 4:32–8. doi: 10.1902/annals.1999.4.1.32 10863373

[42] Qasim SSB Al-Otaibi D Al-Jasser R Gul SS Zafar MS. An Evidence-Based Update on the Molecular Mechanisms Underlying Periodontal Diseases. Int J Mol Sci (2020) 21. doi: 10.3390/ijms21113829 PMC7312805 32481582

[43] Harris ED. Rheumatoid Arthritis. Pathophysiology and Implications for Therapy. N Engl J Med (1990) 322:1277–89. doi: 10.1056/NEJM199005033221805 2271017

[44] Guo Q Wang Y Xu D Nossent J Pavlos NJ Xu J. Rheumatoid Arthritis: Pathological Mechanisms and Modern Pharmacologic Therapies. Bone Res (2018) 6:1–14. doi: 10.1038/s41413-018-0016-9 29736302 PMC5920070

[45] Fang Q Zhou C Nandakumar KS. Molecular and Cellular Pathways Contributing to Joint Damage in Rheumatoid Arthritis. Mediators Inflamm (2020) 2020:3830212. doi: 10.1155/2020/3830212 32256192 PMC7103059

[46] Firestein GS. Evolving Concepts of Rheumatoid Arthritis. Nature (2003) 423:356–61. doi: 10.1038/nature01661 12748655

[47] Rodan GA Martin TJ. Therapeutic Approaches to Bone Diseases. Science (2000) 289:1508–14. doi: 10.1126/science.289.5484.1508 10968781

[48] Zhang J Xie Z Zhang N Zhong J. Nanosuspension Drug Delivery System: Preparation, Characterization, Postproduction Processing, Dosage Form, and Application. In: Nanostructures for Drug Delivery. Shanghai: Elsevier Inc. (2017). p. 413–43. doi: 10.1016/B978-0-323-46143-6.00013-0

[49] Werner SR Morgan JA. Controlling Selectivity and Enhancing Yield of Flavonoid Glycosides in Recombinant Yeast. Bioprocess Biosyst Eng (2010) 33:863–71. doi: 10.1007/s00449-010-0409-7 20148267

[50] Welch AA Hardcastle AC. The Effects of Flavonoids on Bone. Curr Osteoporos Rep (2014) 12:205–10. doi: 10.1007/s11914-014-0212-5 24671371

[51] Santos E Maia B Ferriani A Teixeira S. Flavonoids: Classification, Biosynthesis and Chemical Ecology. Brazil: InTechOpen (2017). doi: 10.5772/67861

[52] Tapas A Sakarkar DM Kakde R. Flavonoids as Nutraceuticals: A Review. Trop J Pharm Res (2008) 7(3):1089–99. doi: 10.4314/tjpr.v7i3.14693

[53] Corradini E Foglia P Giansanti P Gubbiotti R Samperi R Laganà A. Flavonoids: Chemical Properties and Analytical Methodologies of Identification and Quantitation in Foods and Plants. Natural Prod Res (2011) 25:469–95. doi: 10.1080/14786419.2010.482054 21391112

[54] Silva MP Alves JSF Siqueira EM de Souza Neto MA Abreu LS Tavares JF. Isolation and Identification of the Five Novel Flavonoids from Genipa americana Leaves. Molecules (2018) 23(10):2521. doi: 10.3390/molecules23102521 PMC6222654 30279336

[55] Havsteen BH. The Biochemistry and Medical Significance of the Flavonoids. Pharmacol Ther (2002) 96:67–202. doi: 10.1016/S0163-7258(02)00298-X 12453566

[56] Kandaswami C Middleton EJ. Free Radical Scavenging and Antioxidant Activity of Plant Flavonoids. Adv Exp Med Biol (1994) 366:351–76. doi: 10.1007/978-1-4615-1833-4_25 7771265

[57] Hollman PC Katan MB. Dietary Flavonoids: Intake, Health Effects and Bioavailability. Food Chem Toxicol (1999) 37:937–42. doi: 10.1016/S0278-6915(99)00079-4 10541448

[58] Ko K-P. Isoflavones: Chemistry, Analysis, Functions and Effects on Health and Cancer. Asian Pac J Cancer Prev (2014) 15:7001–10. doi: 10.7314/APJCP.2014.15.17.7001 25227781

[59] Sharma V Ramawat KG. Isoflavonoids BT - Natural Products: Phytochemistry, Botany and Metabolism of Alkaloids, Phenolics and Terpenes. Ramawat KG Mérillon J-M, editors. Berlin Heidelberg: Springer (2013) p. 1849–65. doi: 10.1007/978-3-642-22144-6_61

[60] Křížová L Dadáková K Kašparovská J Kašparovský T. Isoflavones. Molecules (2019) 24:1076–104. doi: 10.3390/molecules24061076 PMC6470817 30893792

[61] Kumar P Ahamad T Mishra DP Khan MF. Plant Neoflavonoids: Chemical Structures and Biological Functions BT - Plant-Derived Bioactives: Chemistry and Mode of Action. Swamy MK, editor. The Gateway, Singapore: Springer Singapore (2020) p. 35–57. doi: 10.1007/978-981-15-2361-8_3

[62] Kawser Hossain M Abdal Dayem A Han J Yin Y Kim K Kumar Saha S. Molecular Mechanisms of the Anti-Obesity and Anti-Diabetic Properties of Flavonoids. Int J Mol Sci (2016) 17:569. doi: 10.3390/ijms17040569 27092490 PMC4849025

[63] Bernatoniene J Kopustinskiene DM. The Role of Catechins in Cellular Responses to Oxidative Stress. Molecules (2018) 23:965. doi: 10.3390/molecules23040965 PMC6017297 29677167

[64] Ruiz-Cruz S. Flavonoids: Important Biocompounds in Food. Chaparro-Hernández S, editor. Sonora, México: IntechOpen (2017). Ch. 16. doi: 10.5772/67864

[65] Rozmer Z Perjési P. Naturally Occurring Chalcones and Their Biological Activities. Phytochem Rev (2016) 15:87–120. doi: 10.1007/s11101-014-9387-8

[66] Wong SK Chin KY Ima-Nirwana S. Quercetin as an Agent for Protecting the Bone: A Review of the Current Evidence. Int J Mol Sci (2020) 21:1–37. doi: 10.3390/ijms21176448 PMC7503351 32899435

[67] Braun KF Ehnert S Freude T Egaña JT Schenck TL Buchholz A. Quercetin Protects Primary Human Osteoblasts Exposed to Cigarette Smoke Through Activation of the Antioxidative Enzymes HO-1 and SOD-1. TheScientificWorldJournal (2011) 11:2348–57. doi: 10.1100/2011/471426 PMC3236410 22203790

[68] Wong RWK Rabie ABM. Effect of Quercetin on Bone Formation. J Orthop Res (2008) 26:1061–6. doi: 10.1002/jor.20638 18383168

[69] Prouillet C Mazière JC Mazière C Wattel A Brazier M Kamel S. Stimulatory Effect of Naturally Occurring Flavonols Quercetin and Kaempferol on Alkaline Phosphatase Activity in MG-63 Human Osteoblasts Through ERK and Estrogen Receptor Pathway. Biochem Pharmacol (2004) 67:1307–13. doi: 10.1016/j.bcp.2003.11.009 15013846

[70] Kim YJ Bae YC Suh KT Jung JS. Quercetin, a Flavonoid, Inhibits Proliferation and Increases Osteogenic Differentiation in Human Adipose Stromal Cells. Biochem Pharmacol (2006) 72:1268–78. doi: 10.1016/j.bcp.2006.08.021 16996034

[71] Tsuji M Yamamoto H Sato T Mizuha Y Kawai Y Taketani Y. Dietary Quercetin Inhibits Bone Loss Without Effect on the Uterus in Ovariectomized Mice. J Bone Miner Metab (2009) 27:673–81. doi: 10.1007/s00774-009-0088-0 19495926

[72] Yamaguchi M Weitzmann MN. Quercetin, a Potent Suppressor of NF-κB and Smad Activation in Osteoblasts. Int J Mol Med (2011) 28:521–5. doi: 10.3892/ijmm.2011.749 21769418

[73] Woo J-T Nakagawa H Notoya M Yonezawa T Udagawa N Lee I-S. Quercetin Suppresses Bone Resorption by Inhibiting the Differentiation and Activation of Osteoclasts. Biol Pharm Bull (2004) 27:504–9. doi: 10.1248/bpb.27.504 15056855

[74] Kim HR Kim BM Won JY Lee KA Ko HM Kang YS. Quercetin, a Plant Polyphenol, Has Potential for the Prevention of Bone Destruction in Rheumatoid Arthritis. J Med Food (2019) 22:152–61. doi: 10.1089/jmf.2018.4259 30596535

[75] Guo C Yang RJ Jang K Zhou XL Liu YZ. Protective Effects of Pretreatment With Quercetin Against Lipopolysaccharide-Induced Apoptosis and the Inhibition of Osteoblast Differentiation *via* the MAPK and Wnt/β-Catenin Pathways in MC3T3-E1 Cells. Cell Physiol Biochem (2017) 43:1547–61. doi: 10.1159/000481978 29035884

[76] Luo Z Liang W Luo Z Ge S Li M Du J. Oral Administration of Quercetin Inhibits Bone Loss in Rat Model of Diabetic Osteopenia. Eur J Pharmacol (2011) 670:317–24. doi: 10.1016/j.ejphar.2011.08.014 21914440

[77] Messer JG Hopkins RG Kipp DE. Quercetin Metabolites Up-Regulate the Antioxidant Response in Osteoblasts Isolated From Fetal Rat Calvaria. J Cell Biochem (2015) 116:1857–66. doi: 10.1002/jcb.25141 25716194

[78] Wong SK Chin KY Ima-Nirwana S. The Osteoprotective Effects of Kaempferol: The Evidence From *In Vivo* and *In Vitro* Studies. Drug Des Dev Ther (2019) 13:3497–514. doi: 10.2147/DDDT.S227738 PMC6789172 31631974

[79] Trivedi R Kumar S Kumar A Siddiqui JA Swarnkar G Gupta V. Kaempferol has Osteogenic Effect in Ovariectomized Adult Sprague-Dawley Rats. Mol Cell Endocrinol (2008) 289:85–93. doi: 10.1016/j.mce.2008.02.027 18400372

[80] Zhu J Tang H Zhang Z Zhang Y Qiu C Zhang L. Kaempferol Slows Intervertebral Disc Degeneration by Modifying LPS-Induced Osteogenesis/Adipogenesis Imbalance and Inflammation Response in BMSCs. Int Immunopharmacol (2017) 43:236–42. doi: 10.1016/j.intimp.2016.12.020 28043032

[81] Zhao J Wu J Xu B Yuan Z Leng Y Min J. Kaempferol Promotes Bone Formation in Part *via* the mTOR Signaling Pathway. Mol Med Rep (2019) 20:5197–207. doi: 10.3892/mmr.2019.10747 PMC6854588 31638215

[82] Pang JL Ricupero DA Huang S Fatma N Singh DP Romero JR. Differential Activity of Kaempferol and Quercetin in Attenuating Tumor Necrosis Factor Receptor Family Signaling in Bone Cells. Biochem Pharmacol (2006) 71:818–26. doi: 10.1016/j.bcp.2005.12.023 16434028

[83] Imran M Rauf A Shah ZA Saeed F Imran A Arshad MU. Chemo-Preventive and Therapeutic Effect of the Dietary Flavonoid Kaempferol: A Comprehensive Review. Phytother Res (2019) 33:263–75. doi: 10.1002/ptr.6227 30402931

[84] Nepal M Li L Cho HK Park JK Soh Y. Kaempferol Induces Chondrogenesis in ATDC5 Cells Through Activation of ERK/BMP-2 Signaling Pathway. Food Chem Toxicol (2013) 62:238–45. doi: 10.1016/j.fct.2013.08.034 23989061

[85] Chiou WF Lee CH Liao JF Chen CC. 8-Prenylkaempferol Accelerates Osteoblast Maturation Through Bone Morphogenetic Protein-2/P38 Pathway to Activate Runx2 Transcription. Life Sci (2011) 88:335–42. doi: 10.1016/j.lfs.2010.12.009 21163272

[86] Guo AJ Choi RC Zheng KY Chen VP Dong TT Wang ZT. Kaempferol as a Flavonoid Induces Osteoblastic Differentiation *via* Estrogen Receptor Signaling. Chin Med (2012) 7:1–7. doi: 10.1186/1749-8546-7-10 22546174 PMC3350445

[87] Choi EM. Kaempferol Protects MC3T3-E1 Cells Through Antioxidant Effect and Regulation of Mitochondrial Function. Food Chem Toxicol (2011) 49:1800–5. doi: 10.1016/j.fct.2011.04.031 21565246

[88] Kim CJ. The Effects of Kaempferol-Inhibited Autophagy on Osteoclast Formation. Int J Mol Sci (2018) 19:1–13. doi: 10.3390/ijms19010125 PMC5796074 29301320

[89] Ming LG Chen KM Xian CJ. Functions and Action Mechanisms of Flavonoids Genistein and Icariin in Regulating Bone Remodeling. J Cell Physiol (2013) 228:513–21. doi: 10.1002/jcp.24158 22777826

[90] Wang Z Wang D Yang D Zhen W Zhang J Peng S. The Effect of Icariin on Bone Metabolism and its Potential Clinical Application. Osteoporos Int (2018) 29:535–44. doi: 10.1007/s00198-017-4255-1 29110063

[91] Zhao J Ohba S Shinkai M Chung U Nagamune T. Icariin Induces Osteogenic Differentiation *In Vitro* in a BMP- and Runx2-Dependent Manner. Biochem Biophys Res Commun (2008) 369:444–8. doi: 10.1016/j.bbrc.2008.02.054 18295595

[92] Fu S Yang L Hong H Zhang R. Wnt/β-Catenin Signaling is Involved in the Icariin Induced Proliferation of Bone Marrow Mesenchymal Stem Cells. J Tradit Chin Med (2016) 36:360–8. doi: 10.1016/S0254-6272(16)30050-4 27468552

[93] Hsieh TP Sheu SY Sun JS Chen MH Liu MH. Icariin Isolated From Epimedium Pubescens Regulates Osteoblasts Anabolism Through BMP-2, SMAD4, and Cbfa1 Expression. Phytomedicine (2010) 17:414–23. doi: 10.1016/j.phymed.2009.08.007 19747809

[94] Wu Y Xia L Zhou Y Xu Y Jiang X. Icariin Induces Osteogenic Differentiation of Bone Mesenchymal Stem Cells in a MAPK-Dependent Manner. Cell Prolif (2015) 48:375–84. doi: 10.1111/cpr.12185 PMC6496185 25867119

[95] Xu Q Chen G Liu X Dai M Zhang B. Icariin Inhibits RANKL-Induced Osteoclastogenesis *via* Modulation of the NF-κB and MAPK Signaling Pathways. Biochem Biophys Res Commun (2019) 508:902–6. doi: 10.1016/j.bbrc.2018.11.201 30538045

[96] Huang J Bao Y Xiang W Jing X Guo J Yao X. Icariin Regulates the Bidirectional Differentiation of Bone Marrow Mesenchymal Stem Cells Through Canonical Wnt Signaling Pathway. Evid Based Complement Alternat Med (2017) 2017:1–12. doi: 10.1155/2017/8085325 PMC5763109 29445413

[97] Jing X Du T Chen K Guo J Xiang W Yao X. Icariin Protects Against Iron Overload-Induced Bone Loss *via* Suppressing Oxidative Stress. J Cell Physiol (2019) 234:10123–37. doi: 10.1002/jcp.27678 30387158

[98] Ma HP Ma XN Ge BF Zhen P Zhou J Gao YH. Icariin Attenuates Hypoxia-Induced Oxidative Stress and Apoptosis in Osteoblasts and Preserves Their Osteogenic Differentiation Potential *In Vitro* . Cell Prolif (2014) 47:527–39. doi: 10.1111/cpr.12147 PMC6496789 25355404

[99] Hsieh TP Sheu SY Sun JS Chen MH. Icariin Inhibits Osteoclast Differentiation and Bone Resorption by Suppression of MAPKs/NF-κB Regulated HIF-1α and PGE2 Synthesis. Phytomedicine (2011) 18:176–85. doi: 10.1016/j.phymed.2010.04.003 20554188

[100] Huang J Wu C Tian B Zhou X Ma N Qian Y. Myricetin Prevents Alveolar Bone Loss in an Experimental Ovariectomized Mouse Model of Periodontitis. Int J Mol Sci (2016) 17:1–13. doi: 10.3390/ijms17030422 PMC4813273 27011174

[101] Gupta G Siddiqui MA Khan MM Ajmal M Ahsan R Rahaman MA. Current Pharmacological Trends on Myricetin. Drug Res (2020) 70:448–54. doi: 10.1055/a-1224-3625 32877951

[102] Fu YX Wang YH Tong XS Gong Z Sun XM Yuan JC. EDACO, a Derivative of Myricetin, Inhibits the Differentiation of Gaoyou Duck Embryonic Osteoclasts *In Vitro* . Br Poult Sci (2019) 60:169–75. doi: 10.1080/00071668.2018.1564239 30722674

[103] Gulsoy Z Yucel Z Guven B Balli U. Investigation of Oxidative Stress in Experimental Periodontitis Treated With Myricetin. Ann Med Res (2020) 27:3272. doi: 10.5455/annalsmedres.2020.02.124

[104] Wu C Wang W Tian B Liu X Qu X Zhai Z. Myricetin Prevents Titanium Particle-Induced Osteolysis *In Vivo* and Inhibits RANKL-Induced Osteoclastogenesis *In Vitro* . Biochem Pharmacol (2015) 93:59–71. doi: 10.1016/j.bcp.2014.10.019 25449599

[105] Ying X Chen X Wang T Zheng W Chen L Xu Y. Possible Osteoprotective Effects of Myricetin in STZ Induced Diabetic Osteoporosis in Rats. Eur J Pharmacol (2020) 866:172805. doi: 10.1016/j.ejphar.2019.172805 31756333

[106] Pan X Chen T Zhang Z Chen X Chen C Chen L. Activation of Nrf2/HO-1 Signal With Myricetin for Attenuating ECM Degradation in Human Chondrocytes and Ameliorating the Murine Osteoarthritis. Int Immunopharmacol (2019) 75:105742. doi: 10.1016/j.intimp.2019.105742 31325727

[107] Ko SY. Myricetin Suppresses LPS-Induced MMP Expression in Human Gingival Fibroblasts and Inhibits Osteoclastogenesis by Downregulating NFATc1 in RANKL-Induced RAW 264.7 Cells. Arch Oral Biol (2012) 57:1623–32. doi: 10.1016/j.archoralbio.2012.06.012 22795564

[108] Lee KH Choi EM. Myricetin, a Naturally Occurring Flavonoid, Prevents 2-Deoxy-D-Ribose Induced Dysfunction and Oxidative Damage in Osteoblastic MC3T3-E1 Cells. Eur J Pharmacol (2008) 591:1–6. doi: 10.1016/j.ejphar.2008.06.004 18599037

[109] Ying X Chen X Feng Y Xu HZ Chen H Yu K. Myricetin Enhances Osteogenic Differentiation Through the Activation of Canonical Wnt/β-Catenin Signaling in Human Bone Marrow Stromal Cells. Eur J Pharmacol (2014) 738:22–30. doi: 10.1016/j.ejphar.2014.04.049 24876056

[110] Hsu YL Chang JK Tsai CH Chien TTC Kuo PL. Myricetin Induces Human Osteoblast Differentiation Through Bone Morphogenetic Protein-2/P38 Mitogen-Activated Protein Kinase Pathway. Biochem Pharmacol (2007) 73:504–14. doi: 10.1016/j.bcp.2006.10.020 17113042

[111] Fan S Gao X Chen P Li X. Myricetin Ameliorates Glucocorticoid-Induced Osteoporosis Through the ERK Signaling Pathway. Life Sci (2018) 207:205–11. doi: 10.1016/j.lfs.2018.06.006 29883721

[112] Kuo PL. Myricetin Inhibits the Induction of Anti-Fas IgM-, Tumor Necrosis Factor-α- and Interleukin-1 β-Mediated Apoptosis by Fas Pathway Inhibition in Human Osteoblastic Cell Line MG-63. Life Sci (2005) 77:2964–76. doi: 10.1016/j.lfs.2005.05.026 15982670

[113] Zhu Z Xie W Li Y Zhu Z Zhang W. Effect of Naringin Treatment on Postmenopausal Osteoporosis in Ovariectomized Rats: A Meta-Analysis and Systematic Review. Evid Based Complement Alternat Med (2021) 2021:1–8. doi: 10.1155/2021/6016874 PMC7889366 33628301

[114] Li N Jiang Y Wooley PH Xu Z Yang SY. Naringin Promotes Osteoblast Differentiation and Effectively Reverses Ovariectomy-Associated Osteoporosis. J Orthop Sci (2013) 18:478–85. doi: 10.1007/s00776-013-0362-9 23553541

[115] Liu M Li Y Yang S-T. Effects of Naringin on the Proliferation and Osteogenic Differentiation of Human Amniotic Fluid-Derived Stem Cells. J Tissue Eng Regen Med (2017) 11:276–84. doi: 10.1002/term.1911 24915843

[116] Li F Sun X Ma J Ma X Zhao B Zhang Y. Naringin Prevents Ovariectomy-Induced Osteoporosis and Promotes Osteoclasts Apoptosis Through the Mitochondria-Mediated Apoptosis Pathway. Biochem Biophys Res Commun (2014) 452:629–35. doi: 10.1016/j.bbrc.2014.08.117 25181344

[117] Ang ESM Yang X Chen H Liu Q Zheng MH Xu J. Naringin Abrogates Osteoclastogenesis and Bone Resorption *via* the Inhibition of RANKL-Induced NF-κB and ERK Activation. FEBS Lett (2011) 585:2755–62. doi: 10.1016/j.febslet.2011.07.046 21835177

[118] Hirata M Matsumoto C Takita M Miyaura C Inada M. Naringin Suppresses Osteoclast Formation and Enhances Bone Mass in Mice. J Health Sci (2009) 55:463–7. doi: 10.1248/jhs.55.463

[119] Yu KE Alder KD Morris MT Munger AM Lee I Cahill SV. Re-Appraising the Potential of Naringin for Natural, Novel Orthopedic Biotherapies. Ther Adv Musculoskelet Dis (2020) 12:1–21. doi: 10.1177/1759720X20966135 PMC7727086 33343723

[120] Li C Zhang J Lv F Ge X Li G. Naringin Protects Against Bone Loss in Steroid-Treated Inflammatory Bowel Disease in a Rat Model. Arch Biochem Biophys (2018) 650:22–9. doi: 10.1016/j.abb.2018.05.011 29753722

[121] Wang D Ma W Wang F Dong J Wang D Sun B. Stimulation of Wnt/β-Catenin Signaling to Improve Bone Development by Naringin *via* Interacting With AMPK and Akt. Cell Physiol Biochem (2015) 36:1563–76. doi: 10.1159/000430319 26159568

[122] Wu J-B Fong YC Tsai HY Chen YF Tsuzuki M Tang CH. Naringin-Induced Bone Morphogenetic Protein-2 Expression *via* PI3K, Akt, C-Fos/c-Jun and AP-1 Pathway in Osteoblasts. Eur J Pharmacol (2008) 588:333–41. doi: 10.1016/j.ejphar.2008.04.030 18495116

[123] Kanno SI Shouji A Tomizawa A Hiura T Osanai Y Ujibe M. Inhibitory Effect of Naringin on Lipopolysaccharide (LPS)-Induced Endotoxin Shock in Mice and Nitric Oxide Production in RAW 264.7 Macrophages. Life Sci (2006) 78:673–81. doi: 10.1016/j.lfs.2005.04.051 16137700

[124] Dang ZC Löwik CWGM. The Balance Between Concurrent Activation of ERs and PPARs Determines Daidzein-Induced Osteogenesis and Adipogenesis. J Bone Miner Res (2004) 19:853–61. doi: 10.1359/jbmr.040120 15068509

[125] Jia TL Wang HZ Xie LP Wang XY Zhang RQ. Daidzein Enhances Osteoblast Growth That may be Mediated by Increased Bone Morphogenetic Protein (BMP) Production. Biochem Pharmacol (2003) 65:709–15. doi: 10.1016/S0006-2952(02)01585-X 12628484

[126] de Wilde A Lieberherr M Colin C Pointillart A. A Low Dose of Daidzein Acts as an Erβ-Selective Agonist in Trabecular Osteoblasts of Young Female Piglets. J Cell Physiol (2004) 200:253–62. doi: 10.1002/jcp.20008 15174095

[127] Picherit C Coxam V Bennetau-Pelissero C Kati-Coulibaly S Davicco MJ Lebecque P. Daidzein is More Efficient Than Genistein in Preventing Ovariectomy- Induced Bone Loss in Rats. J Nutr (2000) 130:1675–81. doi: 10.1093/jn/130.7.1675 10867035

[128] Tyagi AM Srivastava K Sharan K Yadav D Maurya R Singh D. Daidzein Prevents the Increase in CD4+CD28null T Cells and B Lymphopoesis in Ovariectomized Mice: A Key Mechanism for Anti-Osteoclastogenic Effect. PloS One (2011) 6:e21216. doi: 10.1371/journal.pone.0021216 21731677 PMC3120851

[129] Jin X Sun J Yu B Wang Y Sun WJ Yang J. Daidzein Stimulates Osteogenesis Facilitating Proliferation, Differentiation, and Antiapoptosis in Human Osteoblast-Like MG-63 Cells *via* Estrogen Receptor–Dependent MEK/ERK and PI3K/Akt Activation. Nutr Res (2017) 42:20–30. doi: 10.1016/j.nutres.2017.04.009 28633868

[130] Hu B Yu B Tang DZ Li SY Wu Y Chen M. Daidzein Promotes Proliferation and Differentiation in Osteoblastic OCT1 Cells *via* Activation of the BMP-2/Smads Pathway. Pharmazie (2017) 72:35–40. doi: 10.1691/ph.2017.6502 29441895

[131] Karieb S Fox SW. Phytoestrogens Directly Inhibit TNF-α-Induced Bone Resorption in RAW264.7 Cells by Suppressing C-Fos-Induced NFATc1 Expression. J Cell Biochem (2011) 112:476–87. doi: 10.1002/jcb.22935 21268069

[132] Kim TH Jung JW Ha BG Hong JM Park EK Kim HJ. The Effects of Luteolin on Osteoclast Differentiation, Function *In Vitro* and Ovariectomy-Induced Bone Loss. J Nutr Biochem (2011) 22:8–15. doi: 10.1016/j.jnutbio.2009.11.002 20233653

[133] Lee JW Ahn JY Hasegawa SI Cha BY Yonezawa T Nagai K. Inhibitory Effect of Luteolin on Osteoclast Differentiation and Function. Cytotechnology (2009) 61:125–34. doi: 10.1007/s10616-010-9253-5 PMC2825295 20162352

[134] Balci Yuce H Toker H Yildirim A Tekin MB Gevrek F Altunbas N. The Effect of Luteolin in Prevention of Periodontal Disease in Wistar Rats. J Periodontol (2019) 90:1481–9. doi: 10.1002/JPER.18-0584 31115905

[135] Nash LA Sullivan PJ Peters SJ Ward WE. Rooibos Flavonoids, Orientin and Luteolin, Stimulate Mineralization in Human Osteoblasts Through the Wnt Pathway. Mol Nutr Food Res (2015) 59:443–53. doi: 10.1002/mnfr.201400592 25488131

[136] Choi EM. Modulatory Effects of Luteolin on Osteoblastic Function and Inflammatory Mediators in Osteoblastic MC3T3-E1 Cells. Cell Biol Int (2007) 31:870–7. doi: 10.1016/j.cellbi.2007.01.038 17368935

[137] Quan H Dai X Liu M Wu C Wang D. Luteolin Supports Osteogenic Differentiation of Human Periodontal Ligament Cells. BMC Oral Health (2019) 19:1–10. doi: 10.1186/s12903-019-0926-y 31655580 PMC6815369

[138] Yang H Liu Q Ahn JH Kim SB Kim YC Sung SH. Luteolin Downregulates IL-1β-Induced MMP-9 and-13 Expressions in Osteoblasts *via* Inhibition of ERK Signalling Pathway. J Enzyme Inhib Med Chem (2012) 27:261–6. doi: 10.3109/14756366.2011.587415 21679050

[139] Chen CY Peng WH Tsai KD Hsu SL. Luteolin Suppresses Inflammation-Associated Gene Expression by Blocking NF-κB and AP-1 Activation Pathway in Mouse Alveolar Macrophages. Life Sci (2007) 81:1602–14. doi: 10.1016/j.lfs.2007.09.028 PMC7094354 17977562

[140] Abbasi N Khosravi A Aidy A Shafiei M. Biphasic Response to Luteolin in MG-63 Osteoblast-Like Cells Under High Glucose-Induced Oxidative Stress. Iran J Med Sci (2016) 41:118–25. PMC4764961 26989282

[141] Dixon RA Ferreira D. Genistein. Phytochemistry (2002) 60:205–11. doi: 10.1016/S0031-9422(02)00116-4 12031439

[142] Albertazzi P Steel SA Bottazzi M. Effect of Pure Genistein on Bone Markers and Hot Flushes. Climacteric (2005) 8:371–9. doi: 10.1080/13697130500345257 16390772

[143] Anderson JJB Ambrose WW Garner SC. Biphasic Effects of Genistein on Bone Tissue in the Ovariectomized, Lactating Rat Model. Proc Soc Exp Biol Med (1998) 217:345–50. doi: 10.3181/00379727-217-44243 9492346

[144] Li YQ Xing XH Wang H Weng XL Bin YS Dong GY. Dose-Dependent Effects of Genistein on Bone Homeostasis in Rats’ Mandibular Subchondral Bone. Acta Pharmacol Sin (2012) 33:66–74. doi: 10.1038/aps.2011.136 22120966 PMC4010271

[145] Fanti P Monier-Faugere MC Geng Z Schmidt J Morris PE Cohen D. The Phytoestrogen Genistein Reduces Bone Loss in Short-Term Ovariectomized Rats. Osteoporos Int (1998) 8:274–81. doi: 10.1007/s001980050065 9797913

[146] Kim M Lim J Lee J-H Lee K-M Kim S Park KW. Understanding the Functional Role of Genistein in the Bone Differentiation in Mouse Osteoblastic Cell Line MC3T3-E1 by RNA-Seq Analysis. Sci Rep (2018) 8:1:1–12. doi: 10.1038/s41598-018-21601-9 29459627 PMC5818530

[147] Heim M Frank O Kampmann G Sochocky N Pennimpede T Fuchs P. The Phytoestrogen Genistein Enhances Osteogenesis and Represses Adipogenic Differentiation of Human Primary Bone Marrow Stromal Cells. Endocrinology (2004) 145:848–59. doi: 10.1210/en.2003-1014 14605006

[148] Lu R Zheng Z Yin Y Jiang Z. Genistein Prevents Bone Loss in Type 2 Diabetic Rats Induced by Streptozotocin. Food Nutr Res (2020) 64:1–12. doi: 10.29219/fnr.v64.3666 PMC7778425 33447176

[149] Liao QC Xiao ZS Qin YF Zhou HH. Genistein Stimulates Osteoblastic Differentiation *via* P38 MAPK-Cbfa1 Pathway in Bone Marrow Culture. Acta Pharmacol Sin (2007) 28:1597–602. doi: 10.1111/j.1745-7254.2007.00632.x 17883946

[150] Pan W Quarles LD Song LH Yu YH Jiao C Tang HB. Genistein Stimulates the Osteoblastic Differentiation *via* NO/cGMP in Bone Marrow Culture. J Cell Biochem (2005) 94:307–16. doi: 10.1002/jcb.20308 15526288

[151] Trzeciakiewicz A Habauzit V Mercier S Barron D Urpi-Sarda M Manach C. Molecular Mechanism of Hesperetin-7-O-Glucuronide, the Main Circulating Metabolite of Hesperidin, Involved in Osteoblast Differentiation. J Agric Food Chem (2010) 58:668–75. doi: 10.1021/jf902680n 19921838

[152] Trzeciakiewicz A Habauzit V Mercier S Lebecque P Davicco M Coxam V. Hesperetin Stimulates Differentiation of Primary Rat Osteoblasts Involving the BMP Signalling Pathway. J Nutr Biochem (2010) 21:424–31. doi: 10.1016/j.jnutbio.2009.01.017 19427185

[153] Kim SY Lee JJCJY Park YD Kang KL Lee JJCJY Heo JS. Hesperetin Alleviates the Inhibitory Effects of High Glucose on the Osteoblastic Differentiation of Periodontal Ligament Stem Cells. PloS One (2013) 8:1–11. doi: 10.1371/journal.pone.0067504 PMC3696082 23840726

[154] Horcajada MN Habauzit V Trzeciakiewicz A Morand C Gil-Izquierdo A Mardon J. Hesperidin Inhibits Ovariectomized-Induced Osteopenia and Shows Differential Effects on Bone Mass and Strength in Young and Adult Intact Rats. J Appl Physiol (2021) 104:648–54. doi: 10.1152/japplphysiol.00441.2007 18174393

[155] Habauzit V Maria S Gil-izquierdo A Trzeciakiewicz A Morand C Barron D. Differential Effects of Two Citrus Fl Avanones on Bone Quality in Senescent Male Rats in Relation to Their Bioavailability and Metabolism. Bone (2011) 49:1108–16. doi: 10.1016/j.bone.2011.07.030 21820093

[156] Goto T Hagiwara K Shirai N Yoshida K Hagiwara H. Apigenin Inhibits Osteoblastogenesis and Osteoclastogenesis and Prevents Bone Loss in Ovariectomized Mice. Cytotechnology (2015) 67:357–65. doi: 10.1007/s10616-014-9694-3 PMC4329293 24500394

[157] Jung WW. Protective Effect of Apigenin Against Oxidative Stress-Induced Damage in Osteoblastic Cells. Int J Mol Med (2014) 33:1327–34. doi: 10.3892/ijmm.2014.1666 24573323

[158] Choi EM. Apigenin Increases Osteoblastic Differentiation and Inhibits Tumor Necrosis Factor-α-Induced Production of Interleukin-6 and Nitric Oxide in Osteoblastic MC3T3-E1 Cells. Pharmazie (2007) 62:216–20. doi: 10.1691/ph.2007.3.6629 17416199

[159] Lee JH Zhou HY Cho SY Kim YS Lee YS Jeong CS. Anti-Inflammatory Mechanisms of Apigenin: Inhibition of Cyclooxygenase-2 Expression, Adhesion of Monocytes to Human Umbilical Vein Endothelial Cells, and Expression of Cellular Adhesion Molecules. Arch Pharm Res (2007) 30:1318–27. doi: 10.1007/BF02980273 18038911

[160] Zhang X Wang G Gurley EC Zhou H. Flavonoid Apigenin Inhibits Lipopolysaccharide-Induced Inflammatory Response Through Multiple Mechanisms in Macrophages. PloS One (2014) 9:1–18. doi: 10.1371/journal.pone.0107072 PMC4156420 25192391

[161] Zhang X Zhou C Zha X Xu Z Li L Liu Y. Apigenin Promotes Osteogenic Differentiation of Human Mesenchymal Stem Cells Through JNK and P38 MAPK Pathways. Mol Cell Biochem (2015) 407:41–50. doi: 10.1007/s11010-015-2452-9 25994505

[162] Zhang Y Zeng X Zhang L Zheng X. Stimulatory Effect of Puerarin on Bone Formation Through Activation of PI3K/Akt Pathway in Rat Calvaria Osteoblasts. Planta Med (2007) 73:341–7. doi: 10.1055/s-2007-967168 17443435

[163] Zhou Y Lian H Liu K Wang D Xiu X Sun Z. Puerarin Improves Graft Bone Defect Through microRNA-155-3p-Mediated P53/TNF-α/STAT1 Signaling Pathway. Int J Mol Med (2020) 46:239–51. doi: 10.3892/ijmm.2020.4595 PMC7255454 32377717

[164] Liu L Liu L Bo T Li S Zhu Z Cui R. Puerarin Suppress Apoptosis of Human Osteoblasts *via* ERK Signaling Pathway. Int J Endocrinol (2013) 2013:1–6. doi: 10.1155/2013/786574 PMC3694486 23843790

[165] Zhang G Wang Y Tang G Ma Y. Puerarin Inhibits the Osteoclastogenesis by Inhibiting RANKL-Dependent and –Independent Autophagic Responses. BMC Complement Altern Med (2019) 19:269. doi: 10.1186/s12906-019-2691-5 31615565 PMC6794871

[166] Xiao L Zhong M Huang Y Zhu J Tang W Li D. Puerarin Alleviates Osteoporosis in the Ovariectomy-Induced Mice by Suppressing Osteoclastogenesis *via* Inhibition of TRAF6/ROS-Dependent MAPK/NF-κB Signaling Pathways. Aging (2020) 12:21706–29. doi: 10.18632/aging.103976 PMC7695364 33176281

[167] Nagaoka M Maeda T Moriwaki S Nomura A Kato Y Niida S. Petunidin, a B-Ring 50-O-Methylated Derivative of Delphinidin, Stimulates Osteoblastogenesis and Reduces Srankl-Induced Bone Loss. Int J Mol Sci (2019) 20:2795. doi: 10.3390/ijms20112795 PMC6600628 31181661

[168] Kaczmarczyk-Sedlak I Wojnar W Zych M Ozimina-Kamińska E Taranowicz J Siwek A. Effect of Formononetin on Mechanical Properties and Chemical Composition of Bones in Rats With Ovariectomy-Induced Osteoporosis. Evid Based Complement Alternat Med (2013) 2013:1–10. doi: 10.1155/2013/457052 PMC3666393 23762138

[169] Singh KB Dixit M Dev K Maurya R Singh D. Formononetin, a Methoxy Isoflavone, Enhances Bone Regeneration in a Mouse Model of Cortical Bone Defect. Br J Nutr (2017) 117:1511–22. doi: 10.1017/S0007114517001556 28689509

[170] Soundharrajan I Kim DH Kuppusamy P Choi KC. Modulation of Osteogenic and Myogenic Differentiation by a Phytoestrogen Formononetin *via* P38mapk-Dependent JAK-STAT and Smad-1/5/8 Signaling Pathways in Mouse Myogenic Progenitor Cells. Sci Rep (2019) 9:1–12. doi: 10.1038/s41598-019-45793-w 31243298 PMC6594940

[171] Huh JE Lee WI Kang JW Nam D Choi DY Park DS. Formononetin Attenuates Osteoclastogenesis *via* Suppressing the RANKL-Induced Activation of NF-κB, C-Fos, and Nuclear Factor of Activated T-Cells Cytoplasmic 1 Signaling Pathway. J Natural Prod (2014) 77:2423–31. doi: 10.1021/np500417d 25397676

[172] Huh JE Kwon NH Baek YH Lee JD Choi DY Jingushi S. Formononetin Promotes Early Fracture Healing Through Stimulating Angiogenesis by Up-Regulating VEGFR-2/Flk-1 in a Rat Fracture Model. Int Immunopharmacol (2009) 9:1357–65. doi: 10.1016/j.intimp.2009.08.003 19695348

[173] Wang Y Bai S Cheng Q Zeng Y Xu X Guan G. Naringenin Promotes Sdf-1/Cxcr4 Signaling Pathway in Bmscs Osteogenic Differentiation. Folia Histochem Cytobiol (2021) 59:66–73. doi: 10.5603/FHC.a2021.0008 33704767

[174] Quan GH Wang H Cao J Zhang Y Wu D Peng Q. Calycosin Suppresses RANKL-Mediated Osteoclastogenesis Through Inhibition of MAPKs and NF-κB. Int J Mol Sci (2015) 16:29496–507. doi: 10.3390/ijms161226179 PMC4691122 26690415

[175] Folwarczna J Zych M Trzeciak HI. Effects of Curcumin on the Skeletal System in Rats. Pharmacol Rep (2010) 62:900–9. doi: 10.1016/S1734-1140(10)70350-9 21098873

[176] Rohanizadeh R Deng Y Verron E. Therapeutic Actions of Curcumin in Bone Disorders. Bonekey Rep (2016) 5:793. doi: 10.1038/bonekey.2016.20 26962450 PMC4774085

[177] Li H Yue L Xu H Li N Li J Zhang Z. Curcumin Suppresses Osteogenesis by Inducing miR-126a-3p and Subsequently Suppressing the WNT/LRP6 Pathway. Aging (Albany NY) (2019) 11:6983–98. doi: 10.18632/aging.102232 PMC6756869 31480018

[178] Dong J Tao L Abourehab M Z H-I. Modified Nanoparticles for Combo-Delivery of Curcumin and Alendronate: Fabrication, Characterization, and Cellular and Molecular Evidences of Enhanced Bone. (2018). Elsevier. doi: 10.1016/j.ijbiomac.2018.05.116 29782984

[179] Tiwari S Agarwal S Seth B Yadav A. Curcumin-Loaded Nanoparticles Potently Induce Adult Neurogenesis and Reverse Cognitive Deficits in Alzheimer’s Disease Model *via* Canonical Wnt/β-Catenin. ACS Publ (2013) 8:76–103. doi: 10.1021/nn405077y 24467380

[180] Tiwari SK Agarwal S Tripathi A Chaturvedi RK. Bisphenol-A Mediated Inhibition of Hippocampal Neurogenesis Attenuated by Curcumin *via* Canonical Wnt Pathway. Mol Neurobiol (2016) 53:3010–29. doi: 10.1007/s12035-015-9197-z 25963729

[181] Vallée A Lecarpentier Y Vallée JN. Curcumin: A Therapeutic Strategy in Cancers by Inhibiting the Canonical WNT/β-Catenin Pathway. J Exp Clin Cancer Res (2019) 38:323. doi: 10.1186/s13046-019-1320-y 31331376 PMC6647277

[182] Dou H Shen R Tao J Huang L Shi H Chen H. Curcumin Suppresses the Colon Cancer Proliferation by Inhibiting Wnt/β-Catenin Pathways *via* miR-130a. Front Pharmacol (2017) 8. doi: 10.3389/fphar.2017.00877 PMC5705620 29225578

[183] Ashrafizadeh M Ahmadi Z Mohamamdinejad R Yaribeygi H Serban M-C Orafai HM. Curcumin Therapeutic Modulation of the Wnt Signaling Pathway. Curr Pharm Biotechnol (2020) 21:1006–15. doi: 10.2174/1389201021666200305115101 32133961

[184] Zhang F Lu C Xu W Shao J Wu L Lu Y. Curcumin Raises Lipid Content by Wnt Pathway in Hepatic Stellate Cell. J Surg Res (2016) 200:460–6. doi: 10.1016/j.jss.2015.08.040 26414021

[185] Prasad C Rath G Mathur S Bhatnagar D Ralhan R. Potent Growth Suppressive Activity of Curcumin in Human Breast Cancer Cells: Modulation of Wnt/β-Catenin Signaling. Chem Biol Interact (2009) 181:263–71.doi: 10.1016/j.cbi.2009.06.012 19573523

[186] Zhang Z Chen H Xu C Song L Huang L Lai Y. Curcumin Inhibits Tumor Epithelial-Mesenchymal Transition by Downregulating the Wnt Signaling Pathway and Upregulating NKD2 Expression in Colon Cancer. Oncol Rep (2016) 35:2615–23. span doi: 10.3892/or.2016.4669 PMC4811403 26985708

[187] Ryu M-J Cho M Song J-Y Yun Y-S Choi I-W Kim D-E. Natural Derivatives of Curcumin Attenuate the Wnt/β-Catenin Pathway Through Down-Regulation of the Transcriptional Coactivator P300. Biochem Biophys Res Commun (2008) 377:1304–8. doi: 10.1016/j.bbrc.2008.10.171 19000900

[188] Notoya M Nishimura H Woo JT Nagai K Ishihara Y Hagiwara H. Curcumin Inhibits the Proliferation and Mineralization of Cultured Osteoblasts. Eur J Pharmacol (2006) 534:55–62. doi: 10.1016/j.ejphar.2006.01.028 16476424

[189] Yamaguchi M Moore TW Sun A Snyder JP Shoji M. Novel Curcumin Analogue UBS109 Potently Stimulates Osteoblastogenesis and Suppresses Osteoclastogenesis: Involvement in Smad Activation and NF-κB Inhibition. Integr Biol (2012) 4:905–13. doi: 10.1039/c2ib20045g 22751853

[190] Oh S Kyung TW Choi HS. Curcumin Inhibits Osteoclastogenesis by Decreasing Receptor Activator of Nuclear factor-kappaB Ligand (RANKL) in Bone Marrow Stromal Cells. Mol Cells (2008) 26(5):486–9. 18719352

[191] Lin S-Y Kang L Chen J Wang C. Phytomedicine H. Molecules (–)-Epigallocatechin-3-Gallate (EGCG) Enhances Osteogenic Differentiation of Human Bone Marrow Mesenchymal Stem Cells. Molecules (2018) 23:3221. doi: 10.3390/molecules23123221 PMC6321548 30563251

[192] CH C ML H JK C SH H GJ W. Green Tea Catechin Enhances Osteogenesis in a Bone Marrow Mesenchymal Stem Cell Line. Osteoporos Int (2005) 16:2039–45. doi: 10.1007/s00198-005-1995-0 16170444

[193] Sun W Frost B Liu J. Oleuropein, Unexpected Benefits! Oncotarget (2017) 8:17409. doi: 10.18632/oncotarget.15538 28407695 PMC5392257

[194] MM T H BY O K F G H T. Evaluation of the Effect of Oleuropein on Alveolar Bone Loss, Inflammation, and Apoptosis in Experimental Periodontitis. J Periodontal Res (2019) 54:624–32. doi: 10.1111/jre.12662 31032945

[195] Woźniak M Michalak B Wyszomierska J Dudek MK Kiss AK. Effects of Phytochemically Characterized Extracts From Syringa Vulgaris and Isolated Secoiridoids on Mediators of Inflammation in a Human Neutrophil Model. Front Pharmacol (2018) 9:349. doi: 10.3389/fphar.2018.00349 29695965 PMC5904404

[196] Feng Z Li X Lin J Zheng W Hu Z Xuan J. Oleuropein Inhibits the IL-1β-Induced Expression of Inflammatory Mediators by Suppressing the Activation of NF-κB and MAPKs in Human Osteoarthritis Chondrocytes. Food Funct (2017) 8:3737–44. doi: 10.1039/C7FO00823F 28952621

[197] Castejón ML Rosillo MÁ Montoya T González-Benjumea A Fernández-Bolaños JG Alarcón-de-la-Lastra C. Oleuropein Down-Regulated IL-1β-Induced Inflammation and Oxidative Stress in Human Synovial Fibroblast Cell Line SW982. Food Funct (2017) 8:1890–8. doi: 10.1039/C7FO00210F 28426090

[198] FilipRPossemiersSHeyerickAPinheiroIRaszewskiGDaviccoMJ. Twelve-Month Consumption of a Polyphenol Extract From Olive (Olea Europaea) in a Double Blind, Randomized Trial Increases Serum Total Osteocalcin Levels and Improves Serum Lipid Profiles in Postmenopausal Women With Osteopenia. J Nutr Health Aging (2015) 19:77–86. doi: 10.1007/s12603-014-0480-x 25560820

[199] Fujita K Otsuka T Yamamoto N Kainuma S Ohguchi R Kawabata T. (-)-Epigallocatechin Gallate But Not Chlorogenic Acid Upregulates Osteoprotegerin Synthesis Through Regulation of Bone Morphogenetic Protein-4 in Osteoblasts. Exp Ther Med (2017) 14:417–23. doi: 10.3892/etm.2017.4491 PMC5488595 28672948

[200] Torre E Iviglia G Cassinelli C Morra M. "Potentials of Polyphenols in Bone-Implant Devices", in Polyphenols. InTech (2018). pp.s 1–28. doi: 10.5772/intechopen.76319

